# Reversal of the T cell immune system reveals the molecular basis for T cell lineage fate determination in the thymus

**DOI:** 10.1038/s41590-022-01187-1

**Published:** 2022-04-29

**Authors:** Miho Shinzawa, E. Ashley Moseman, Selamawit Gossa, Yasuko Mano, Abhisek Bhattacharya, Terry Guinter, Amala Alag, Xiongfong Chen, Maggie Cam, Dorian B. McGavern, Batu Erman, Alfred Singer

**Affiliations:** 1 Experimental Immunology Branch, National Cancer Institute, National Institutes of Health, Bethesda, MD USA; 2 Viral Immunology and Intravital Imaging Section, National Institute of Neurological Disorders and Stroke, National Institutes of Health, Bethesda, MD USA; 3 Department of Immunology, Duke University School of Medicine, Durham, NC USA; 4 Office of Science and Technology Resources, Office of the Director, Center for Cancer Research, National Cancer Institute, National Institutes of Health, Bethesda, MD USA; 5 CCR-SF Bioinformatics Group, Advanced Biomedical Computational Science, Biomedical Informatics and Data Science Directorate, Frederick National Laboratory for Cancer Research, Frederick, MD USA; 6 Department of Molecular Biology and Genetics, Bogazici University, Istanbul, Turkey

**Keywords:** CD4-positive T cells, CD8-positive T cells

## Abstract

T cell specificity and function are linked during development, as MHC-II-specific TCR signals generate CD4 helper T cells and MHC-I-specific TCR signals generate CD8 cytotoxic T cells, but the basis remains uncertain. We now report that switching coreceptor proteins encoded by *Cd4* and *Cd8* gene loci functionally reverses the T cell immune system, generating CD4 cytotoxic and CD8 helper T cells. Such functional reversal reveals that coreceptor proteins promote the helper-lineage fate when encoded by *Cd4*, but promote the cytotoxic-lineage fate when encoded in *Cd8*—regardless of the coreceptor proteins each locus encodes. Thus, T cell lineage fate is determined by *cis*-regulatory elements in coreceptor gene loci and is not determined by the coreceptor proteins they encode, invalidating coreceptor signal strength as the basis of lineage fate determination. Moreover, we consider that evolution selected the particular coreceptor proteins that *Cd4* and *Cd8* gene loci encode to avoid generating functionally reversed T cells because they fail to promote protective immunity against environmental pathogens.

## Abstract (web summary)

To determine how T cell lineage fates are determined in the thymus, Singer and colleagues generated ‘FlipFlop’ mice with a functionally reversed T cell immune system that distinguishes TCR signal strength versus TCR signal duration.

## Main

Development of mature T cells in the thymus is driven by signals transduced by components of the T cell antigen receptor (TCR). Fully assembled αβ TCR complexes are first expressed on immature CD4^+^CD8^+^ (double positive, DP) thymocytes, and these cells are signaled by their TCR to undergo positive selection and to differentiate into mature CD4 or CD8 single positive (SP) T cells^1,2^. It is during positive selection that TCR-signaled DP thymocytes make lineage fate choices and differentiate into SP T cells with either helper or cytotoxic function^2^. Expression of the helper-lineage transcription factor ThPOK directs thymocytes to differentiate into helper T cells^3–6^, whereas expression of the cytotoxic-lineage transcription factor Runx3 directs thymocytes to differentiate into cytotoxic T cells^7–11^. It remains uncertain how TCR-signaled thymocytes are induced to express these lineage-specific factors, making lineage fate determination a critical feature of T cell development that still requires investigation.

T cell specificity and function are linked during thymic selection because MHC-II-specific TCR signals generate CD4 helper T cells and MHC-I-specific TCR signals generate CD8 cytotoxic T cells^12^. However, the molecular basis for this linkage continues to be a major issue of contention, with two main perspectives. The ‘strength of signal’ model attributes T cell lineage fates to differences in TCR/coreceptor signaling strengths, with strong CD4-dependent TCR signals inducing the helper-lineage fate and weak CD8-dependent TCR signals inducing the cytotoxic-lineage fate^13–15^. Although this perspective has been contradicted by a variety of experiments^16–20^, it has never been definitively invalidated. In fact, recent single-cell gene analyses of thymocytes undergoing positive selection are thought to support strength-of-signal as the basis for lineage fate determination^21,22^. The ‘kinetic signaling model’ attributes T cell lineage fates to the opposite effects of TCR signaling on *Cd4* and *Cd8* gene transcription in DP thymocytes^23,24^. TCR signaling upregulates *Cd4* but terminates *Cd8* transcription, causing persistent CD4-dependent TCR signaling and disrupted CD8-dependent TCR signaling during positive selection^24^. In the kinetic signaling perspective, persistent/long-duration TCR signaling induces ThPOK and the helper-lineage fate, while disrupted or short-duration TCR signaling results in Runx3 expression and the cytotoxic-lineage fate^23^. Because strong signals tend to have a long duration and weak signals tend to have a short duration, it has not been possible to unequivocally distinguish the effects of signal duration on lineage fate from those of signal intensity.

We undertook the present study to assess whether thymocyte lineage fate is determined by coreceptor gene loci that regulate TCR signal duration or by coreceptor proteins, which determine TCR signal strength. We constructed unique FlipFlop mice, whose *Cd4* and *Cd8* genes encode the opposite coreceptor proteins of wild-type (WT) mice. We discovered that switching the coreceptor proteins that the *Cd4* and *Cd8* genes encode generates a reversed T cell immune system, with cytotoxic CD4 T cells generated by MHC-II-specific TCR signals (CD4/MHC-II) and CD8 helper T cells generated by MHC-I-specific TCR signals (CD8/MHC-I). Such functional reversal revealed that weakly signaling CD8 coreceptors encoded by *Cd4* gene loci promoted long-duration CD8/MHC-I TCR signaling and the helper-lineage fate, whereas strongly signaling CD4 coreceptors encoded in *Cd8* gene loci promoted short-duration CD4/MHC-II TCR signaling and the cytotoxic-lineage fate. As a result, this study invalidates strength-of-signal as the basis for lineage fate determination and documents that T cell lineage fate is instead determined by *cis*-regulatory elements in coreceptor gene loci that regulate duration of TCR signaling during positive selection. Moreover, assessment of in vivo immune responses in FlipFlop mice revealed that reversed-function T cells fail to promote protective immunity against environmental pathogens, which explains why evolution selected the particular coreceptor proteins that *Cd4* and *Cd8* genes encode in all surviving species.

## Results

### Reversal of lineage factor expression in FlipFlop mice

**Fig. 1:**
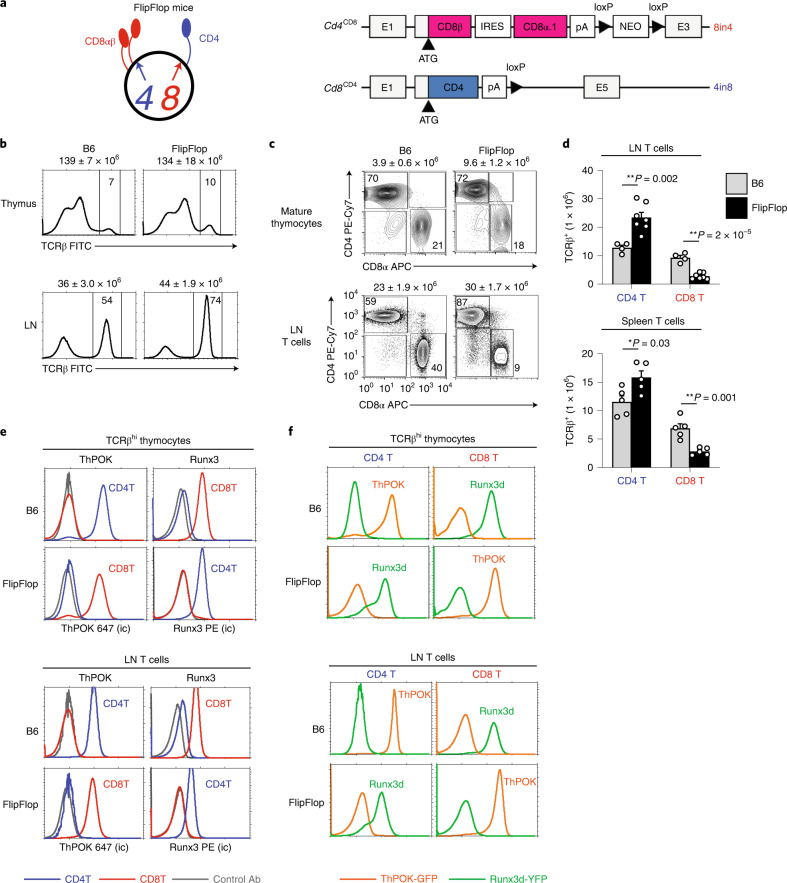
Characterization of FlipFlop mice. **a**, Schematic of altered *Cd4* and *Cd8α* gene loci in FlipFlop mice. Left, surface proteins encoded by altered *Cd4*^CD8^ (*4*) and *Cd8*^CD4^ (*8*) gene loci in FlipFlop DP thymocytes. Right, schematic of the altered *Cd4*^CD8^ and *Cd8*^CD4^ gene loci in FlipFlop mice: E, exons; IRES, internal ribosome entry site; pA, polyadenylation signals; NEO, neomycin-resistance cassette. The altered *Cd4*^CD8^ gene locus was obtained from 8in4 mice, which were constructed for this study as described in Extended Data Fig. 1a; the altered *Cd8*^CD4^ gene locus was obtained from 4in8 mice, which were reported previously^25^. **b**, TCRβ expression in the thymus and LN from B6 and FlipFlop mice. Total cell number (mean ± s.e.m) is shown above histograms, and numbers within histograms indicate frequency of TCRβ^hi^ cells (n = 4–7 per strain, representative of 4–7 independent experiments). **c**, CD4 versus CD8α profile of CD24^-^TCRβ^+^ mature thymocytes and TCRβ^+^ LN T cells from B6 and FlipFlop mice. Numbers (mean ± s.e.m) of mature thymocytes and LN T cells is shown above profiles and numbers within profiles indicate frequency of cells in each box (*n* = 4–7 per strain, representative of 4–7 independent experiments). **d**, Numbers of CD4 and CD8 T cells in LN (top) and spleen (bottom) from B6 (gray bar) and FlipFlop (black bar) mice (B6: *n* = 4, FlipFlop: *n* = 7, representative of 4–5 independent experiments). *P < 0.05, **P < 0.01 (two-tailed unpaired *t*-test); mean + s.e.m. **e**, Intracellular staining (ic) of ThPOK and Runx3 of CD4 (blue line) and CD8 (red line) T cells among TCRβ^hi^ thymocytes (top) and TCRβ^+^ LN T cells (bottom) from B6 and FlipFlop mice. Gray lines in histograms indicate staining with control antibody (Ab; *n* = 3 per strain, 3 independent experiments). **f**, ThPOK-GFP (orange line) and Runx3d-YFP (green line) reporter expression in CD4 and CD8 T cells among TCRβ^hi^ thymocytes (top) and TCRβ^+^ LN T cells (bottom) from B6 and FlipFlop mice (*n* = 3 per strain, representative of 3 independent experiments).

**Extended Data Fig. 1:**
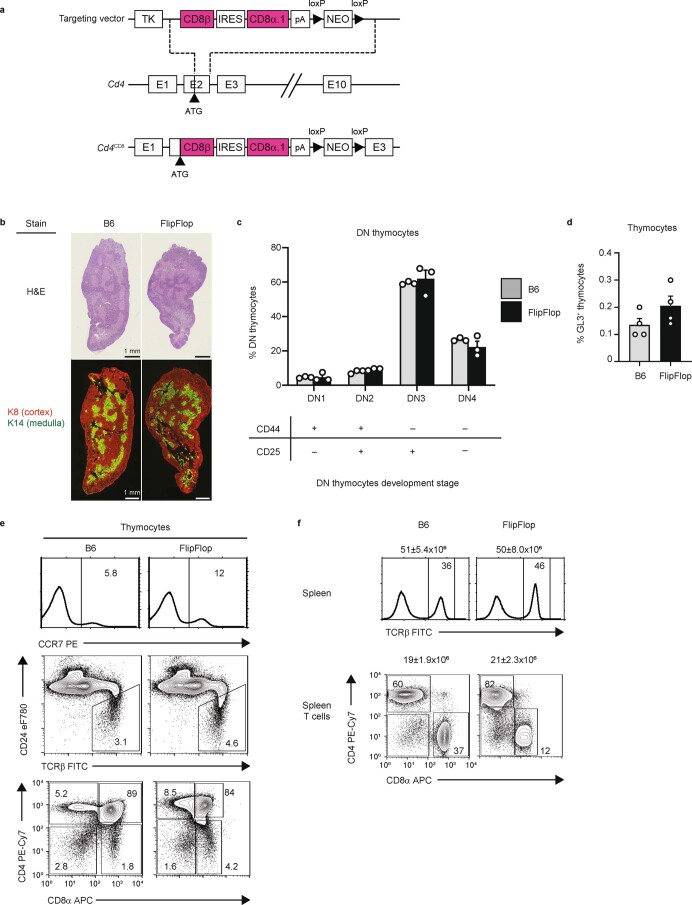
Effect of altered *Cd4* and *Cd8* gene loci on T cell development. (**a**) Alteration of the endogenous *Cd4* gene locus to encode CD8αβ proteins (*Cd4*^CD8^). A targeting vector that consists of a TK cassette, CD8β cDNA, IRES, CD8α.1 cDNA, poly A sequence, and a NEO-resistance cassette flanked by directional loxP sites (top) was homologously recombined into the translation start site in exon 2 of the *Cd4* gene (middle), generating novel mice known as ‘8in4’. Note that the re-engineered *Cd4*^CD8^ gene locus encodes CD8αβ.1 proteins (bottom). (**b**) H&E staining (top) and immunofluorescence staining (bottom) of thymic sections from B6 and FlipFlop mice. Staining of Keratin-8 (K8, red, cortex) and Keratin-14 (K14, green, medulla) is shown. (n=3-4/strain, representative of 2-3 independent experiments). (**c**) DN (CD4^-^CD8^-^) thymocyte development as defined by CD44 and CD25 on lineage-negative thymocytes from B6 (gray bar) and FlipFlop (black bar) mice as indicated. Lineage-negative cells were CD3^-^, B220^-^, CD11b^-^, TER119^-^, Gr-1^-^, CD4^-^, and CD8α^-^ thymocytes. DN1 (CD44^+^CD25^-^), DN2 (CD44^+^CD25^+^), DN3 (CD44^-^CD25^+^), and DN4 (CD44^-^CD25^-^). (n=4/strain, 3 independent experiments). (**d**) Frequency of GL3^+^ γδ T cells in the thymus of B6 (gray bar) and FlipFlop (black bar) mice (n=4/strain, 3 independent experiments). (**e**) CCR7^+^ thymocytes, CD24^-^TCRβ^+^ mature thymocytes, and CD4 vs CD8α profiles from B6 and FlipFlop thymi (n=4-7/strain, representative of 4-5 independent experiments). (**f**) TCRβ expression and CD4 vs CD8 profiles of TCRβ^+^ T cells in spleens from B6 and FlipFlop mice. Total cell number (mean±s.e.m) is shown above histograms and numbers within the profiles indicate frequency of cells within that box (n=4-7/strain, representative of 4-5 independent experiments). Mean+s.e.m. (c and d).

To assess whether thymocyte lineage fate is determined by coreceptor gene loci, rather than the coreceptor proteins they encode, we constructed FlipFlop mice with *Cd4* and *Cd8a* gene loci encoding the opposite CD4 and CD8 coreceptor proteins of WT mice (Fig. 1a left and Extended Data Fig. 1a). As described in the Methods, *Cd8a* gene loci in FlipFlop mice were altered to encode CD4 proteins (*Cd8*^CD4^)^25^, and *Cd4* gene loci were altered to encode CD8αβ.1 proteins (*Cd4*^CD8^), which are distinct from CD8αβ.2 proteins in WT B6 mice^26^ (Fig. 1a right).

Histologic examination revealed that FlipFlop thymi were similar in size and cellularity to B6 thymi, with clearly demarcated cortical and medullary thymic areas identified by keratin-8 and keratin-14 staining, respectively (Extended Data Fig. 1b), so thymic structure was unaffected by the coreceptor proteins that the *Cd4* and *Cd8* gene loci encoded. Differentiation of early DN thymocytes and γδ T cells was also unaffected, as their frequencies were identical in FlipFlop and B6 mice (Extended Data Fig. 1c,d). Positive selection of αβ T cells was then assessed by three criteria: differentiation into TCRβ^hi^ and CCR7^+^ thymocytes, generation of mature SP thymocytes, and appearance of CD4 and CD8 TCRβ^+^ T cells in the periphery (Fig. 1b–d and Extended Data Fig. 1e,f). All three assessments revealed that αβ T cells in FlipFlop mice were positively selected into both CD4 and CD8 SP T cells, but with more CD4 and fewer CD8 T cells in the lymph node (LN) and spleen than in B6 mice, partly because of reduced expansion of peripheral FlipFlop CD8 T cells (Fig. 1c,d).

Positively selected CD4 and CD8 FlipFlop T cells were assessed for expression of the helper-lineage transcription factor ThPOK and the cytotoxic-lineage transcription factor Runx3. Remarkably, ThPOK and Runx3 expression in FlipFlop T cells were opposite of that in B6 T cells, as FlipFlop CD8 T cells expressed the helper-lineage factor ThPOK and FlipFlop CD4 T cells expressed the cytotoxic-lineage factor Runx3 (Fig. 1e). To independently confirm these findings, FlipFlop T cells were also assessed for the lineage reporter genes ThPOK-GFP and Runx3d-YFP. In contrast to B6 T cells, FlipFlop CD4 T cells expressed the cytotoxic-lineage reporter gene Runx3d-YFP and FlipFlop CD8 T cells expressed the helper-lineage reporter gene ThPOK-GFP (Fig. 1f). Thus, switching the coreceptor proteins that *Cd4* and *Cd8* gene loci encode reverses the lineage factors that CD4 and CD8 T cells express. Consequently, the lineage factors that CD4 and CD8 T cells express depend on which coreceptor proteins *Cd4* and *Cd8* gene loci encode.

**Fig. 2:**
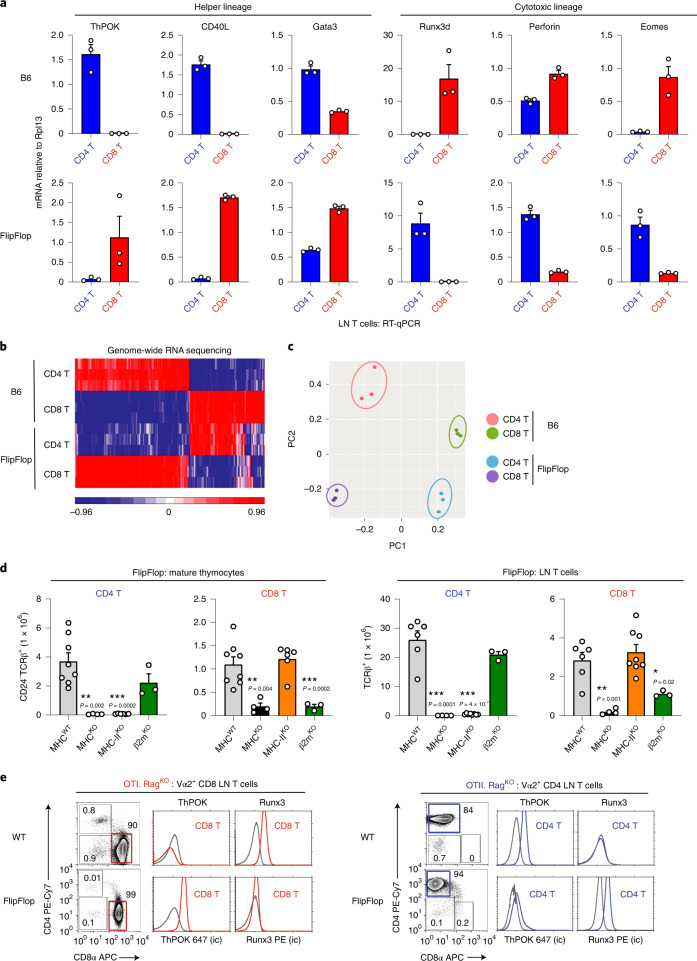
Reversed lineage fate of FlipFlop T cells. **a**, RT–qPCR analysis of CD4 (blue bar) and CD8 (red bar) TCRβ^+^ LN T cells from B6 and FlipFlop mice. Results are normalized to the control gene *Rpl13* (*n* = 4 per strain, representative of 3 independent experiments with technical triplicates). **b**, RNA-sequencing analysis of CD4 and CD8 LN T cells from B6 and FlipFlop mice; 335 genes that are differentially expressed between B6 CD4 and CD8 LN T cells were evaluated in the heat map for expression in FlipFlop LN T cells (*n* = 3 per group, *P* < 0.0001, fivefold change). **c**, Principal component analysis (PCA) from RNA-sequencing analysis in **b**. **d**, Numbers of CD4 and CD8 T cells among CD24^–^TCRβ^+^ mature FlipFlop thymocytes and TCRβ^+^ FlipFlop LN T cells in mice with the indicated MHC deficiencies. (WT: thymocytes *n* = 8, LN T cells *n* = 6, MHC^KO^: *n* = 4, MHC-II^KO^: *n* = 8, β2m^KO^: *n* = 3, 3–9 independent experiments). *P < 0.05, **P < 0.01, ***P < 0.001 (two-tailed unpaired *t*-test). Mean + s.e.m. **e**, CD4 versus CD8α profiles and intracellular staining (ic) of ThPOK and Runx3 of Vα2^+^ LN T cells from Rag^KO^ WT and Rag^KO^ FlipFlop mice expressing monoclonal OT-I (left) and OT-II (right) transgenic TCR. Gray lines in histograms indicate staining with control antibody. Numbers within profiles indicate frequency of cells in each box (*n* = 3–6/strain, representative of 2–4 independent experiments).

We next asked whether coreceptor proteins that *Cd4* and *Cd8* gene loci encode affect only lineage factor expression, or whether they affect expression of other helper- and cytotoxic-lineage genes as well. Quantitative reverse transcription–PCR (RT–qPCR) analyses revealed that FlipFlop CD8 T cells expressed the helper-lineage genes encoding CD40L^27,28^ and GATA-3 (ref. ^29^) in addition to ThPOK, and that FlipFlop CD4 T cells expressed the cytotoxic-lineage genes encoding perforin^30^ and Eomes^31^ in addition to Runx3d (Fig. 2a). We expanded our analysis by performing genome-wide RNA sequencing, which identified 335 genes in B6 mice that were differentially expressed in CD4 helper-lineage T cells compared with CD8 cytotoxic-lineage T cells (Fig. 2b). Heat-map assessment of gene expression revealed that FlipFlop CD4 T cells closely resembled B6 CD8 cytotoxic-lineage T cells, while FlipFlop CD8 T cells closely resembled B6 CD4 helper-lineage T cells (Fig. 2b). The few exceptions in FlipFlop T cells are due to strain 129 genes^32^ remaining in FlipFlop mice from the 129R1 embryonic stem cell line that was originally used to generate *Cd8*^CD4^ gene loci^25^. Principal component analysis confirmed primary similarities between FlipFlop CD4 and B6 CD8 T cells, and between FlipFlop CD8 and B6 CD4 T cells (Fig. 2c). Together, these data show that, in FlipFlop mice, CD4 T cells are cytotoxic-lineage cells and CD8 T cells are helper-lineage cells. We conclude that, regardless of which coreceptor proteins they encode, *Cd4* gene loci regulate expression of helper-lineage genes and *Cd8* gene loci regulate expression of cytotoxic-lineage genes. As a result, switching the coreceptor proteins that *Cd4* and *Cd8* gene loci encode functionally reverses the lineage fate of CD4- and CD8-expressing T cells so that the FlipFlop immune system consists of CD4 cytotoxic-lineage T cells and CD8 helper-lineage T cells.

### *Cd4* and *Cd8* gene loci promote different lineage factors

Because CD4 and CD8 T cells express opposite lineage factors and acquire opposite lineage fates in FlipFlop compared with B6 mice, we examined their MHC recognition specificities. We found that CD8 T cell generation was impaired by MHC-I deficiency in FlipFlop.β2m^KO^ and FlipFlop.MHC^KO^ mice, and that CD4 T cell generation was impaired by MHC-II deficiency in FlipFlop.MHC-II^KO^ and FlipFlop.MHC^KO^ mice, which indicated that FlipFlop CD8 T cells are generated by MHC-I-specific selection and that FlipFlop CD4 T cells are generated by MHC-II-specific selection (Fig. 2d). Thus, the MHC recognition specificity of CD4 and CD8 T cells in FlipFlop mice is the same as that in B6 mice, indicating that MHC-I and MHC-II recognition by CD8 and CD4 coreceptor proteins is unaffected by the coreceptor gene loci in which they are encoded.

**Extended Data Fig. 2:**
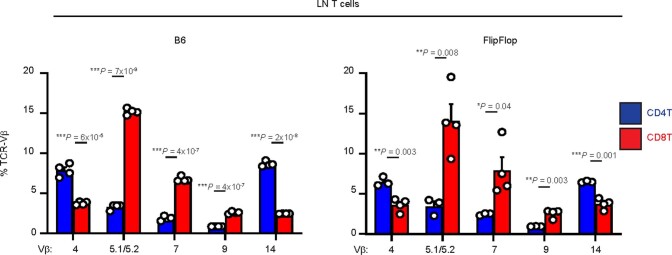
TCR-Vβ expression of FlipFlop T cells. Frequency of TCR-Vβ 4, 5.1/5.2, 7, 9, and 14 in LN T cells from B6 (left) and FlipFlop (right) mice (B6: n=4, FlipFlop: n=3, 3-4 independent experiments). TCR-Vβs expressed differently (>2-fold changes) between B6 CD4 and CD8 T cells were analyzed in FlipFlop T cells. FlipFlop CD4 and CD8 T cells were obtained from β2m^KO^ and MHC-II^KO^ mice, respectively. * P <0.05, ** P<0.01, *** P<0.001 (two-tailed unpaired *t*-test). Mean+s.e.m.

We then compared TCR-Vβ usage by CD4 and CD8 T cells in B6 and FlipFlop mice as a potential way to observe TCR repertoire shifts in FlipFlop and B6 T cells because of their opposite lineage fates. We identified TCR-Vβ proteins whose usage significantly differed between CD4/MHC-II and CD8/MHC-I T cells in B6 mice and found that their usage also significantly differed between CD4/MHC-II and CD8/MHC-I FlipFlop T cells (Extended Data Fig. 2). Thus TCR-Vβ usage was similar in FlipFlop and B6 T cells, despite their opposite lineage fates.

Next, we examined positive selection and lineage fate determination by monoclonal OT-I and OT-II transgenic TCRs in Rag^KO^ FlipFlop and Rag^KO^ WT mice. MHC-I-specific OT-I TCR selected CD8 T cells and MHC-II-specific OT-II TCR selected CD4 T cells in both FlipFlop and WT mice (Fig. 2e), confirming that the MHC specificity of CD8 and CD4 T cell positive selection is identical in FlipFlop and WT mice. In contrast, lineage factor expression is opposite in FlipFlop and WT mice, as MHC-I-restricted OT-I CD8 T cells expressed ThPOK in FlipFlop mice but expressed Runx3 in WT mice (Fig. 2e left). Similarly, MHC-II-restricted OT-II CD4 T cells expressed Runx3 in FlipFlop mice but expressed ThPOK in WT mice (Fig. 2e right). These results document that monoclonal T cells signaled by identical TCRs and coreceptor proteins express opposite lineage factors in FlipFlop and WT mice. Consequently, both CD4 and CD8 T cells express the helper-lineage factor ThPOK when their coreceptor protein is encoded by *Cd4* but express the cytotoxic-lineage factor Runx3 when their coreceptor protein is encoded in *Cd8*. We conclude that, regardless of which coreceptor protein *Cd4* and *Cd8* genes encode, *Cd4* gene loci promote ThPOK expression and the helper-lineage fate, whereas *Cd8* gene loci promote Runx3 expression and the cytotoxic-lineage fate.

### Effect of gene loci on coreceptor protein expression

**Fig. 3:**
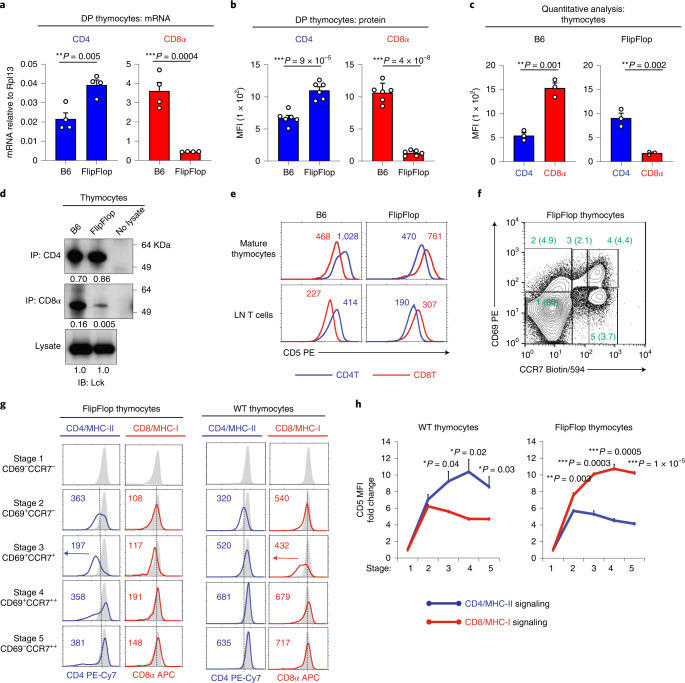
Coreceptor expression on thymocytes undergoing positive selection. **a**, RT–qPCR analysis of CD4 and CD8α mRNAs of pre-selection (CD69^−^CCR7^−^) DP thymocytes (*n* = 4 per strain, representative of 2 independent experiments with technical triplicates). **b**, Mean fluorescence intensity (MFI) of CD4 and CD8α on DP thymocytes. Staining histograms are shown in Extended Data Fig. 3a (*n* = 6 per strain, 6 independent experiments). **c**, Quantitative analysis of CD4 and CD8α on B6 and FlipFlop thymocytes. Thymocytes were stained to saturation with either rat anti-mouse CD4 or rat anti-mouse CD8α unconjugated IgG monoclonal antibodies and then were secondarily stained with anti-rat IgG FITC-conjugated antibody. Staining histograms are shown in Extended Data Fig. 3b (*n* = 3 per strain, 3 independent experiments). **d**, Quantitation of Lck bound to CD4 and CD8α proteins on thymocytes. Thymocyte lysates were immunoprecipitated with anti-CD4 or anti-CD8α antibodies and immunoblotted using anti-Lck antibody. Intensity of Lck bands was normalized to whole lysate, which was set equal to 1.0 (representative of 4 independent experiments). **e**, CD5 expression on CD4 T cells (blue line) and CD8 T cells (red line) among CD24^−^TCRβ^+^ mature thymocytes and TCRβ^+^ LN T cells (*n* = 5 per strain, representative of 5 independent experiments). Numbers in histograms show MFI. **f**, Development of TCR-signaled FlipFlop thymocytes. CD69 versus CCR7 profile identifies five sequential stages (1–5) of positive selection, with TCR-unsignaled cells being stage 1 and TCR-signaled cells developing sequentially into stages 2–5. Numbers in parentheses show frequency of cells at each stage. **g**, Coreceptor kinetics during positive selection. FlipFlop thymocytes (left) and WT thymocytes (right) were assessed for surface CD4 expression during MHC-II selection in β2m^KO^ mice and for surface CD8α expression during MHC-I selection in MHC-II^KO^ mice. Numbers in histograms show MFI. Gray histograms show coreceptor expression on TCR-unsignaled stage 1 thymocytes. Vertical lines mark coreceptor expression levels on TCR-signaled stage 2 thymocytes. (**f**,**g**; *n* = 3−5 per strain, 3–4 independent experiments). **h**, Kinetics of CD5 expression on WT and FlipFlop thymocytes during positive selection. CD5 MFI was normalized to stage 1 thymocytes, which were set as 1.0 (*n* = 3 per strain, 2 independent experiments). *P < 0.05, **P < 0.01, ***P < 0.001 (two-tailed unpaired *t*-test); mean ± s.e.m. (**a**–**c** and **h**). Source data

**Extended Data Fig. 3:**
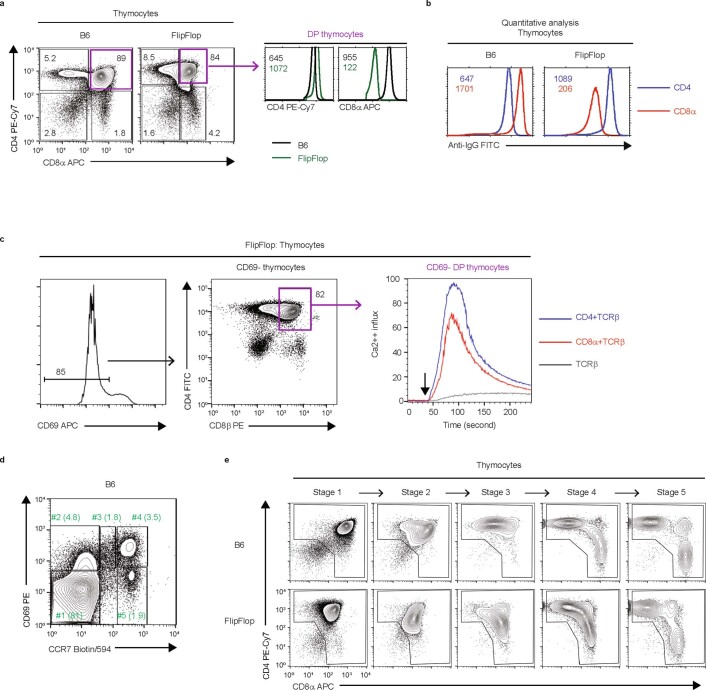
Stages of positive selection in the thymus. (**a**) CD4 vs CD8α expression on whole thymocytes (left) and DP thymocytes (right) from B6 and FlipFlop mice. Numbers within profiles indicate frequency of cells in each box (left) and numbers in histograms indicate MFI (right). (n=7/strain, representative of 7 independent experiments). (**b**) Quantitative analysis of CD4 and CD8α surface expression on thymocytes from B6 and FlipFlop mice, related to Fig. 3c. Numbers indicate MFI. (**c**) Intracellular calcium mobilization stimulated by TCR/coreceptor signaling of DP FlipFlop thymocytes. Thymocytes were stimulated with anti-TCRβ biotin conjugated antibody either alone (gray bar) or together with anti-CD4 (blue bar) or anti-CD8α (red bar) biotin conjugated antibodies and CD69^-^ DP thymocytes were analyzed. Black arrow indicates crosslinking by avidin (n=3, representative of 3 independent experiments). (**d**) CD69 vs CCR7 profile of B6 WT thymocytes. Numbers within the profile show frequency of cells in each stage. (**e**) CD4 vs CD8α profile of WT and FlipFlop thymocytes at Stages 1-5 of development as shown in Fig. 3g. As shown in the profile, DN thymocytes were excluded from the analysis.

To understand how *Cd4* and *Cd8* gene loci influence T cell lineage fate, we first examined their regulation of coreceptor expression in FlipFlop thymocytes (Fig. 3a–c and Extended Data Fig. 3a,b). Compared with B6 DP thymocytes, FlipFlop DP thymocytes express more CD4 mRNA and protein and express less CD8 mRNA and protein (Fig. 3a,b and Extended Data Fig. 3a), which indicates that coreceptor transcription is greater from *Cd8* gene loci than from *Cd4* gene loci. Consistent with this finding, quantitative flow cytometric analysis further revealed that FlipFlop thymocytes express substantially more CD4 than CD8 surface coreceptors, while the reverse is true for B6 thymocytes (Fig. 3c and Extended Data Fig. 3b). Thus, *Cd4* and *Cd8* gene loci regulate the amount of coreceptor protein that thymocytes express. Because FlipFlop thymocytes express lower amounts of CD8 surface coreceptors than do B6 thymocytes, they contained even less CD8-associated Lck tyrosine kinase^33,34^ than did B6 thymocytes (0.5% versus 16%) (Fig. 3d). Consequently, CD8 coreceptor signaling would be weaker than CD4 coreceptor signaling in FlipFlop thymocytes than it is in B6 thymocytes. To examine this expectation, we signaled unstimulated DP thymocytes with anti-TCR/coreceptor monoclonal antibodies and assessed calcium mobilization. Because anti-TCR by itself fails to signal DP thymocytes^34^, calcium flux induced by anti-TCR/CD4 monoclonal antibodies and anti-TCR/CD8 monoclonal antibodies reflects the strength of CD4 and CD8 coreceptor signaling. As expected, TCR/CD8 coengagement generated weaker signals than TCR/CD4 coengagement in FlipFlop DP thymocytes (Extended Data Fig. 3c), revealing that CD8 coreceptor signaling is weaker than CD4 coreceptor signaling in FlipFlop thymocytes.

Because CD5 expression levels are thought to reflect strength and/or duration of TCR/coreceptor signaling^35^, we next examined CD5 expression on FlipFlop and B6 T cells in the thymus and periphery. CD5 expression was higher on CD4 than on CD8 T cells in B6 mice, as expected, but we were surprised to find that CD5 expression in FlipFlop mice was reversed and was higher on CD8 than on CD4 FlipFlop T cells (Fig. 3e). Because CD8 signaling is weaker in FlipFlop than in B6 mice because of their very low CD8 surface expression and their exceptionally low amounts of CD8-associated Lck (Fig. 3a–d and Extended Data Fig. 3c), high CD5 expression on FlipFlop CD8 T cells cannot reflect strong TCR/CD8 signaling but might reflect long-duration TCR/CD8 signaling.

### T lineage fate is determined by TCR signal duration

TCR signaling of positive selection transiently terminates *Cd8* gene transcription but not *Cd4* gene transcription, which causes surface expression of *Cd8*-encoded coreceptor proteins to acutely decline but surface expression of *Cd4*-encoded coreceptor proteins to persist. Consequently, *Cd8*-dependent signaling is disrupted while *Cd4*-dependent signaling persists^23,24^. To visualize changes in surface coreceptor protein expression on signaled thymocytes during positive selection, we identified thymocytes at sequential stages of positive selection by expression of CD69 and CCR7 (ref. ^36^). CD69^−^CCR7^−^ cells are stage 1 pre-selection thymocytes; CD69^−^CCR7^+^ cells are stage 2 thymocytes that have just been TCR-signaled to undergo positive selection; and CD69^+^CCR7^+^ and CD69^−^CCR7^+^ cells are TCR-signaled cells at subsequent stages of positive selection (Fig. 3f and Extended Data Fig. 3d,e). Indeed, transient termination of *Cd8* gene transcription caused an acute decline in CD4 expression on stage 3 FlipFlop thymocytes and in CD8 expression on stage 3 WT thymocytes (Fig. 3g). The acute decline in CD4 expression on FlipFlop thymocytes during positive selection would disrupt CD4-dependent MHC-II signaling and cause it to have a short duration, whereas the steady increase in CD8 surface expression on FlipFlop thymocytes would cause CD8-dependent MHC-I signaling to persist and have a long duration (Fig. 3g left). Note that the opposite changes in coreceptor expression and positive selection signaling occurred in WT thymocytes (Fig. 3g right).

To assess signaling during positive selection, we analyzed CD5 expression on TCR-signaled FlipFlop and WT thymocytes at sequential stages of positive selection. We compared CD5 expression on TCR-signaled thymocytes (stages 2–5) with TCR-unsignaled thymocytes (stage 1) whose values were set equal to 1.0, and found that CD5 on TCR-signaled stage 2 thymocytes increased by six- to eightfold in both FlipFlop and WT mice (Fig. 3h). Importantly, CD5 on FlipFlop thymocytes at subsequent stages 3–5 further increased during CD8/MHC-I signaling but declined during CD4/MHC-II signaling, with the opposite occurring in WT thymocytes (Fig. 3h). These findings reveal that, in FlipFlop mice, CD8/MHC-I-signaling is long duration and CD4/MHC-II-signaling is short duration, which is opposite of that in WT mice. Thus, in FlipFlop mice, long-duration CD8/MHC-I signaling induced the helper-lineage fate and high CD5 expression, despite weak CD8 signaling; short-duration CD4/MHC-II signaling induced the cytotoxic-lineage fate and low CD5 expression, despite strong CD4 signaling. Consequently, thymocyte lineage fate and CD5 expression levels reflect TCR signaling duration, not TCR signaling strength.

**Extended Data Fig. 4:**
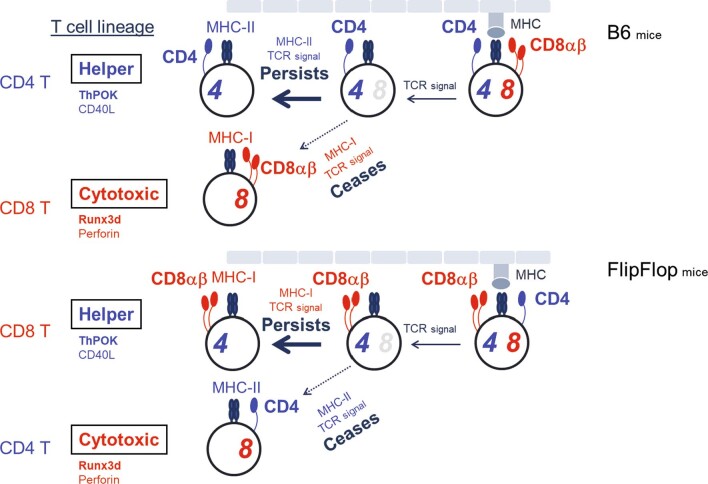
Schematic of the kinetic signaling view of T cell lineage determination in the thymus of B6 and FlipFlop mice. TCR-mediated positive selection signaling of DP thymocytes upregulates *Cd4* but terminates *Cd8* gene transcription which results in increased surface expression of *Cd4*-encoded coreceptor proteins and reduced surface expression of *Cd8*-encoded coreceptor proteins. Consequently, in FlipFlop mice, TCR signaling increases CD8 coreceptor protein expression and reduces CD4 coreceptor protein expression, resulting in persistence/long-duration of CD8/MHC-I TCR signaling and disruption/short-duration of CD4/MHC-II TCR signaling in FlipFlop thymocytes. Thus CD8/MHC-I signaled FlipFlop thymocytes differentiate into helper-lineage T cells, while CD4/MHC-II signaled FlipFlop thymocytes differentiate into cytotoxic-lineage T cells.

We conclude that coreceptor proteins encoded in the *Cd4* gene locus promote persistent and long-duration positive-selection signaling that induces high CD5 expression, ThPOK expression, and the helper-lineage fate, and that coreceptor proteins encoded in the *Cd8* gene locus promote disrupted and short-duration positive selection signaling that stimulates low CD5 expression and allows induction of Runx3 and cytotoxic-lineage fate (schematized in Extended Data Fig. 4). Thus, T cell lineage fate is determined by *Cd4* and *Cd8* gene loci that transcriptionally regulate the kinetics of coreceptor protein expression and determine TCR signaling duration during positive selection.

### A reversed T cell immune system

Switching the coreceptor proteins that *Cd4* and *Cd8* gene loci encode resulted in generation of a reversed T cell immune system, with CD4/MHC-II cytotoxic T cells and CD8/MHC-I helper T cells. To determine the immunocompetence of reversed-function T cells, we examined in vivo immune responses.

**Fig. 4:**
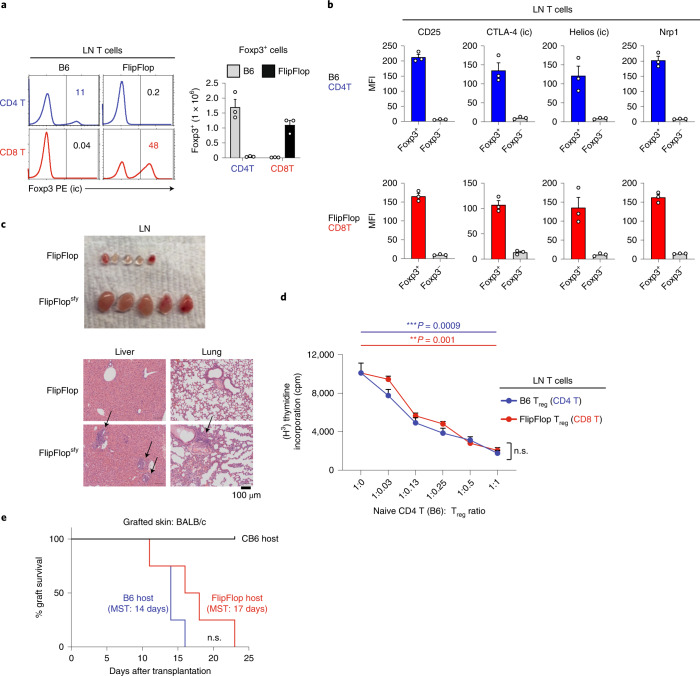
Immunocompetence and self-tolerance in FlipFlop mice. **a**, Intracellular staining for Foxp3 in TCRβ^+^ LN T cells from B6 and FlipFlop mice (left). Numbers in histograms indicate frequency of cells in that box. Numbers (mean ± s.e.m.) of Foxp3^+^ cells among CD4 and CD8 LN T cells in B6 (gray bar) and FlipFlop (black bar) mice are shown in bar graphs (right). (*n* = 5 per strain, representative of 5 independent experiments). **b**, Expression of T_reg_ proteins on T_reg_ cells (Foxp3^+^) and non-T_reg_ cells (Foxp3^–^) among B6 CD4 or FlipFlop CD8 TCRβ^+^ LN T cells (*n* = 5 per strain, representative of 5 independent experiments); mean ± s.e.m. **c**, LNs (top) and H&E stain of tissue sections (bottom) from FlipFlop and FlipFlop^sfy^ male mice (4 weeks old). Arrows indicate regions of lymphocytic cell infiltration (*n* = 4 per strain, representative of 4 independent experiments). **d**, In vitro T_reg_ suppression assay. Purified naive CD4 TCRβ^+^ LN T cells from B6 mice were stimulated in vitro for 3 days with immobilized anti-CD3ε monoclonal antibodies and titrated doses of either B6 CD4 T_reg_ (CD4^+^Foxp3-GFP^+^) or FlipFlop CD8 T_reg_ (CD8^+^Foxp3-GFP^+^) cells sorted from LNs. After 3 days, cultures were assessed for [H^3^]thymidine incorporation (mean ± s.e.m.). Data are representative of three independent experiments. **P < 0.01, ***P < 0.001 (two-tailed unpaired *t*-test); mean + s.e.m. **e**, Skin allograft rejection. Tail skins from BALB/c mice were grafted onto the flanks of indicated host mice. Graph indicates graft survival over time on B6 (blue line, *n* = 8), FlipFlop (red line, *n* = 9), and CB6 (black line, *n* = 4) host mice (log-rank (Mantel–Cox) test, *P* = 0.15 between B6 and FlipFlop host mice, 3 independent experiments). Median survival times (MSTs) on B6 and FlipFlop mice were 14 and 17 days, respectively. n.s., not significant.

**Extended Data Fig. 5:**
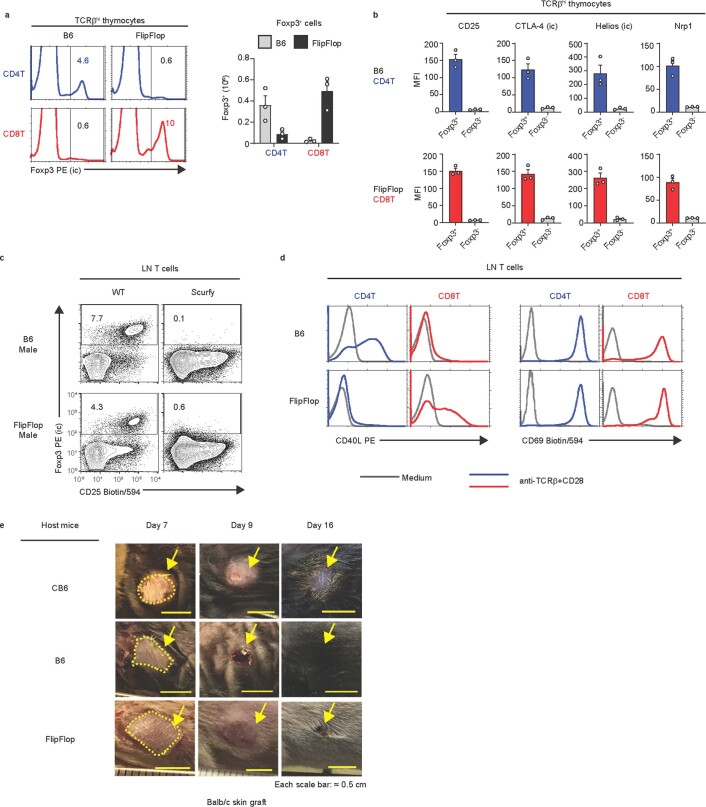
Thymic Tregs and allograft rejection. (**a**) Intracellular staining (ic) of Foxp3 in CD4 and CD8 T cells among TCRβ^hi^ thymocytes from B6 and FlipFlop mice (left). Bar graph shows numbers of Foxp3^+^ T cells. (mean+s.e.m., n=5/strain, representative of 5 independent experiments). (**b**) Treg-associated protein expression in B6 CD4 and FlipFlop CD8 helper-lineage T cells. (mean+s.e.m., n=5/strain, representative of 5 independent experiments). (**c**) Impact of the Scurfy mutation on B6 and FlipFlop LN T cells. (n=3/strain, representative of 3 independent experiments). (**d**) In vitro stimulation of CD40L and CD69 surface expression on B6 and FlipFlop LN T cells by medium (gray bar) or by immobilized anti-TCRβ+CD28 mAbs for 24 h (n=3/strain, 3 independent experiments). (e) Kinetics of skin allograft rejection.

We first considered that species survival requires a functional and self-tolerant T cell immune system, and that self-tolerance requires regulatory T (T_reg_) cells expressing the Foxp3 transcription factor^37^. Because T_reg_ cells in B6 mice are generated from CD4/MHC-II helper-lineage T cells, we were surprised to find that FlipFlop mice generate Foxp3^+^ T cells from CD8/MHC-I helper-lineage T cells and that these CD8 T_reg_ cells display the same expression profiles of CD25, CTLA-4, Helios, and Neuropilin-1 (Nrp-1) proteins as those of B6 CD4 T_reg_ cells^37^ (Fig. 4a,b and Extended Data Fig. 5a,b). To determine whether CD8 T_reg_ cells were important for self-tolerance in FlipFlop mice, we introduced the X-chromosome-linked scurfy (*sfy*) Foxp3 gene mutation into FlipFlop mice^38,39^. In fact, self-tolerance was abrogated in FlipFlop^sfy^ male mice, which lacked T_reg_ cells and developed markedly enlarged lymph nodes and lymphocytic infiltrations into liver and lung (Fig. 4c and Extended Data Fig. 5c). We then further assessed T_reg_ function in vitro and found that FlipFlop CD8 T_reg_ cells were as effective as B6 CD4 T_reg_ cells in suppressing responses of stimulated B6 CD4 responder T cells (Fig. 4d). Thus, CD8 Foxp3^+^ T_reg_ cells maintain self-tolerance in vivo and inhibit T cell activation in vitro.

Species survival also depends on a functional T cell immune system to provide protection against environmental pathogens. To document that peripheral FlipFlop T cells respond to TCR stimulation, we stimulated FlipFlop T cells in vitro with immobilized anti-TCR/CD28 monoclonal antibodies and observed that FlipFlop CD4 and CD8 T cells both upregulated surface CD69 expression (Extended Data Fig. 5d right panels). However, only stimulated helper-lineage T cells upregulated surface CD40L expression, and these were CD8^+^ in FlipFlop mice but CD4^+^ in B6 mice (Extended Data Fig. 5d left panels). As a first test of in vivo T cell function, we assessed rejection of skin allografts^40^. Tailskin grafts from H-2^d^ donor BALB/c mice were placed on fully allogeneic H-2^b^ FlipFlop mice, as well as H-2^b^ B6 recipient mice as a positive control and semi-syngeneic H-2^b/d^ BALB/c × B6 (CB6) mice as a negative control. Unlike CB6 mice, which did not reject BALB/c grafts, FlipFlop mice did reject BALB/c grafts, with a median survival time (MST) of 17 days, which was not significantly prolonged relative to that of B6 mice (*P* = 0.15) (Fig. 4e and Extended Data Fig. 5e). Thus, functionally reversed T cells in FlipFlop mice are immunocompetent to reject skin allografts.

### Humoral response to soluble antigens

**Fig. 5:**
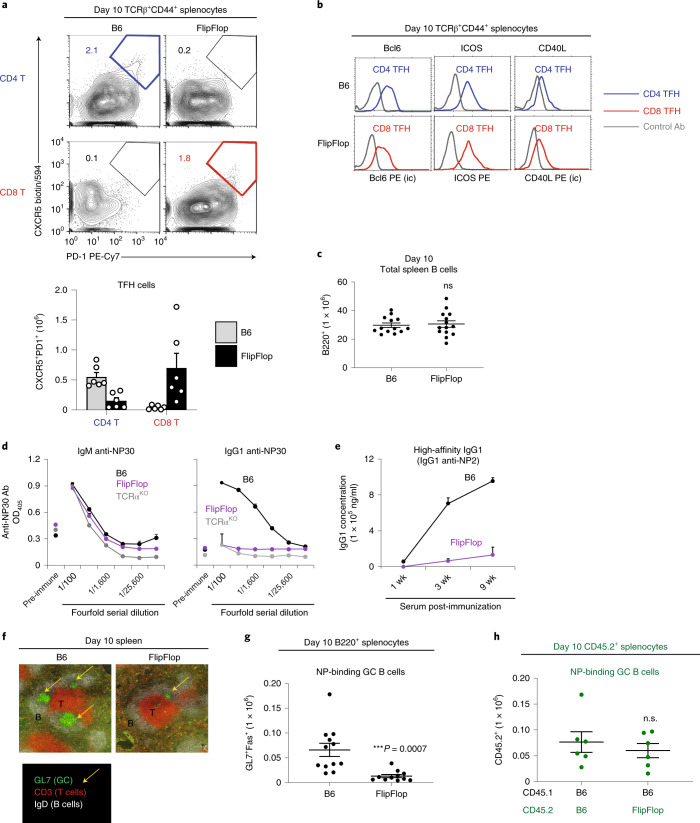
Humoral immune responses in FlipFlop mice. Mice were immunized with NP-KLH/alum by i.p. injection and analyzed. **a**, T_FH_ cells in immunized B6 and FlipFlop mice (day 10). Numbers indicate percentage of CXCR5^+^PD1^+^ T_FH_ cells (top). Bar graph shows T_FH_ (CXCR5^+^PD1^+^) cell numbers (mean ± s.e.m.) among TCRβ^+^CD44^+^ (bottom). (n = 6, representative of 3 independent experiments). Gate for TCRβ^+^CD44^+^ T cells is shown in Extended Data Fig. 6a. **b**, Characterization of T_FH_ cells. T_FH_ cells (TCRβ^+^CD44^+^CXCR5^+^PD1^+^) in FlipFlop and B6 spleens were analyzed on post-immunization day 10 (*n* = 4 per strain, representative of 2–3 independent experiments). **c**, Numbers of B220^+^ B cells in immunized B6 and FlipFlop spleens (day 10) (B6: *n* = 13, FlipFlop: *n* = 14, 7 independent experiments). Staining profile is shown in Extended Data Fig. 6b. **d**, Circulating IgM and IgG1 anti-NP30 antibodies (optical density (OD) at 405 nm) in serum from B6 (black line), FlipFlop (purple line), and TCRα^KO^ (gray line) mice at 1 week post-immunization, measured by ELISA. Pre-immunization serum was included as a negative control (B6 and FlipFlop: *n* = 4, TCRα^KO^: *n* = 3, 3 independent experiments); mean ± s.e.m. **e**, High-affinity IgG1 production. Concentration of IgG1 anti-NP2 antibody (ng/ml) in serum over time after immunization, as measured by ELISA (*n* = 3–4/strain, 3 independent experiments); mean ± s.e.m. **f**, Immunofluorescence staining of spleen sections from immunized B6 and FlipFlop mice (day 10). Staining of GL7 (green and yellow arrow, GC), CD3 (red, T cells), and IgD (white, B cells) is shown (*n* = 3 per strain, representative of 3 independent experiments). **g**, Numbers of NP-binding GC B cells (NP^+^B220^+^GL7^+^Fas^+^) in the spleens of immunized B6 and FlipFlop mice (day 10). Staining profile of immunized splenocytes is shown in Extended Data Fig. 6b (B6: *n* = 12, FlipFlop: *n* = 13, 6–7 independent experiments). **h**, Induction of NP-binding GC B cell induction in mixed BM chimeras from Extended Data Fig. 6c. Mixed BM chimeras reconstituted with both B6 and FlipFlop BM cells were immunized with NP-KLH/alum and were assessed 10 days later for numbers of B6-origin and FlipFlop-origin NP-binding GC B cells. Staining profile and gate are shown in Extended Data Fig. 6d,e (*n* = 6 per strain, 2 independent experiments). *P < 0.05, **P < 0.01, ***P < 0.001 (two-tailed unpaired *t*-test); mean ± s.e.m. (**c**,**g**,**h**).

**Extended Data Fig. 6:**
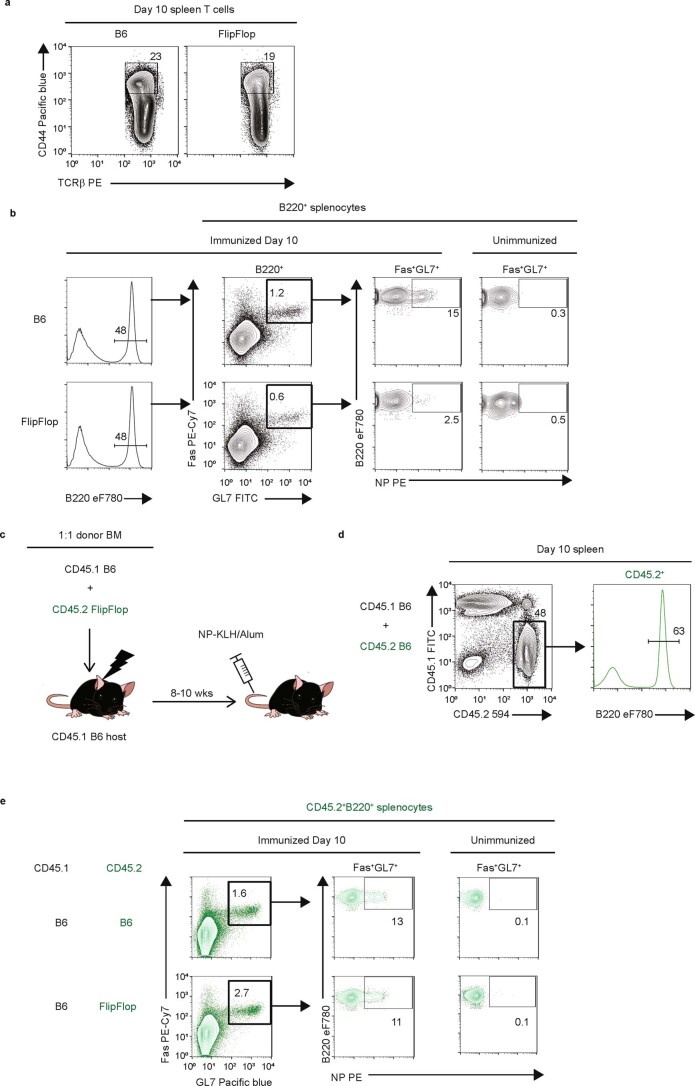
Humoral immune responses to immunization with soluble antigens. (**a**) Gate for TCRβ^+^CD44^+^ T cells in immunized spleen. (**b**) GC B cells and NP-binding GC B cells in the spleen from immunized B6 and FlipFlop mice. GC B cells were identified as B220^+^Fas^+^GL7^+^ cells and those that bound NP were specific for NP. Numbers in profiles indicate frequency of cells in each box (n=8-13/strain, 6-8 independent experiments). (**c**) Experimental model for construction and immunization of mixed BM chimeras shown in Fig. 5h. Partner BM cells (CD45.2) from B6 or FlipFlop mice were mixed with donor BM cells (CD45.1) from B6 mice at a 1:1 ratio and injected into irradiated B6 host mice (CD45.1). Mice were immunized with NP-KLH/Alum 8-10 wks after reconstitution. (**d**) Gate for CD45.2^+^B220^+^ cells in immunized spleen from BM chimeras. (**e**) GC B cells and NP-binding GC B cells in mixed BM chimeras. Fas and GL7 expressions (left), and NP-binding GC B cells (right) among CD45.2^+^B220^+^ splenocytes from immunized (day 10) or unimmunized mixed BM chimeras. Numbers in profiles indicate frequency of cells in each box (n=6/strain, 2 independent experiments).

To assess the in vivo function of CD8/MHC-I helper T cells, we examined the humoral response of FlipFlop mice. T follicular helper (T_FH_) cells are a specialized population of antigen-specific CD4 helper T cells resident in lymphoid organs that interact with antigen-specific B cells and activate them to form germinal centers (GCs) and to secrete IgG antibodies^41,42^. Because CD8 TFH cells have not previously been described, we immunized FlipFlop mice with NP-KLH and then looked for T_FH_ cells, which are CXCR5^+^PD1^+^ T cells. In fact, CD8 T_FH_ cells did arise in immunized FlipFlop mice, and these were generated in comparable numbers to CD4 T_FH_ cells in B6 mice (Fig. 5a and Extended Data Fig. 6a). Moreover, FlipFlop CD8 T_FH_ cells expressed Bcl6, ICOS, and CD40L, as did B6 CD4 T_FH_ cells (Fig. 5b). We then assessed the ability of CD8 T_FH_ cells in FlipFlop mice to promote anti-NP humoral immune responses stimulated by NP-KLH. Notably, although FlipFlop mice and B6 mice had similar numbers of B cells (Fig. 5c), FlipFlop B cells produced only IgM anti-NP antibodies after immunization like T cell-deficient TCRα^KO^ immunized mice (Fig. 5d). FlipFlop mice failed to produce any IgG1 anti-NP antibodies, even at 9 weeks after immunization when B6 mice were producing high amounts of high-affinity IgG1 antibodies (Fig. 5d,e). Thus, CD8 T_FH_ cells failed to activate FlipFlop B cells to undergo T-dependent immunoglobulin (Ig) class switching and Ig affinity maturation.

Histologic examination of FlipFlop spleens revealed that they were virtually devoid of GCs (Fig. 5f green color), even though their T cell follicles (Fig. 5f red color) were located adjacent to surrounding B cell follicles (Fig. 5f white color). The few GC B cells (identified as Fas^+^GL7^+^B220^+^ cells) that were present in FlipFlop spleens were not NP-binding and so were not specific for the immunizing NP antigen (Fig. 5g and Extended Data Fig. 6b). Thus, FlipFlop B cells could not be stimulated to become NP-specific GC B cells by CD8 T_FH_ cells. However, they could be stimulated to do so by CD4 T_FH_ cells of B6 origin in mixed bone marrow (BM) chimeras (Fig. 5h and Extended Data Fig. 6c–e). These results indicate that CD8 T_FH_ cells are specifically unable to productively interact with antigen-specific B cells.

Our finding that CD8/MHC-I TFH cells are generated in immunized FlipFlop mice indicates that dendritic cells (DCs) that the T_FH_ cells interact with can cross-present the immunizing antigen onto their surface MHC-I complexes^43^. However, these CD8/MHC-I T_FH_ cells fail to activate antigen-specific B cells to form GCs or to undergo class switching or affinity maturation, which all indicate that CD8 T_FH_ cells cannot mediate cognate interactions with B cells, likely because B cells cannot cross-present exogenous antigens onto MHC-I surface complexes^44^.

### Immune response to virus infection

**Fig. 6:**
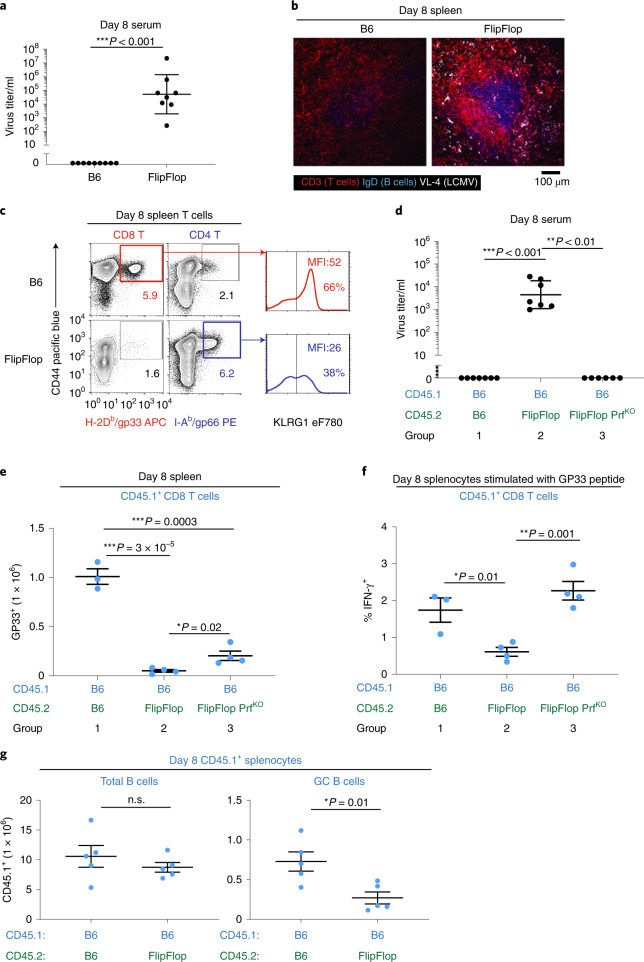
Immune responses to virus infection. Mice were infected with LCMV-Arm (2 × 10^6^ PFU/mouse) by i.v. injection and analyzed on day 8. **a**, Circulating virus titer (FFU: focus forming unit/ml, geometric mean ± s.d.) in the serum on day 8. (B6: *n* = 9, FlipFlop: *n* = 8, 4 independent experiments). **b**, Immunofluorescence staining of spleens on day 8 after infection. Staining with monoclonal antibodies specific for CD3 (red, T cells), IgD (blue, B cells), and VL-4 (white, LCMV) (*n* = 3 per strain, representative of 2 independent experiments). **c**, Virus-specific T cells in B6 and FlipFlop spleens were identified by H-2D^b^/GP33 and I-A^b^/GP66 viral tetramers and contained KLRG1^+^ cells (right) (*n* = 3 per strain, representative of 3 independent experiments). **d**, Virus-infected mixed BM chimeras. Circulating virus titers (FFU/ml, geometric mean ± s.d.) on day 8 after virus infection in the serum of mixed BM chimeras that were generated as in Extended Data Fig. 7b (B6: *n* = 7, FlipFlop: *n* = 7, FlipFlop Prf^KO^: *n* = 6, 2 independent experiments). **e**, FlipFlop cytotoxic T cells prevent in vivo expansion of virus-specific B6 T cells. Graph indicates numbers of B6-origin H-2D^b^/GP33-specific CD8 T cells (CD45.1) in spleens of mixed BM chimeras (day 8). Staining profiles and gate are shown in Extended Data Fig. 7d,e (group 1: *n* = 3, group 2: *n* = 4, group 3: *n* = 4, 2 independent experiments). **f**, In vivo FlipFlop cytotoxic T cells prevent in vitro activation of virus-specific B6 CD8 T cells. In vitro GP33 peptide stimulation of splenocytes from infected mixed BM chimeras (day 8) were assessed for IFNγ^+^ induction of B6-origin CD8 T cells (CD45.1). Staining profiles are shown in Extended Data Fig. 7f (group 1: *n* = 3, group 2: *n* = 4, group 3: *n* = 4, 2 independent experiments). **g**, Impact of FlipFlop T cells on numbers of B cells and GC B cells in the spleen from infected mixed BM chimeras. Spleens from infected mixed BM chimeras (day 8) were assessed for numbers of CD45.1 B6-origin B cells (B220^+^; left) and GC B cells (GL7^+^FAS^+^; right) (*n* = 5/group, 2 independent experiments). *P < 0.05, **P < 0.01, ***P < 0.001 (Mann–Whitney two-tailed unpaired test for **a** and **d**, two-tailed unpaired *t*-test for **e**–**g**); mean ± s.e.m. (**e**–**g**).

**Extended Data Fig. 7:**
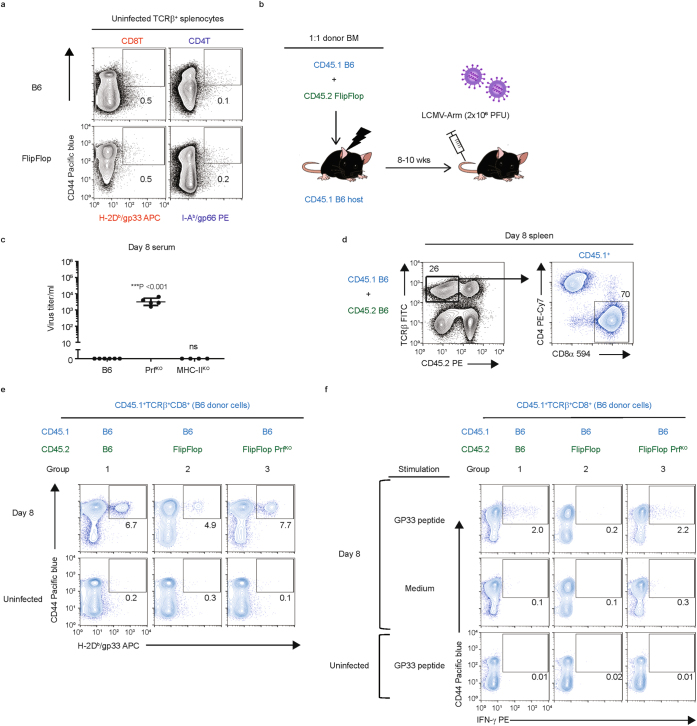
LCMV-Arm infection of FlipFlop mice. (**a**) Detection of virus-specific T cells by viral tetramers. Staining of T cells by H-2D^b^/GP33 and I-A^b^/GP66 viral peptide tetramers in the spleens of uninfected B6 and FlipFlop mice. Numbers in profiles indicate frequency of cells in each box (n=3/strain, representative of 3 independent experiments). (**b**) Experimental model for virus infection of mixed BM chimeras shown in Fig. 6d-g. Partner BM cells (CD45.2) from B6 or FlipFlop mice were mixed with donor BM cells (CD45.1) from B6 mice at a 1:1 ratio and injected into irradiated B6 host mice (CD45.1). Mice were infected with LCMV-Arm 8-10 wks after reconstitution. (**c**) Circulating virus titer (FFU/ml, geometric mean±SD) in the serum of infected mice (day 8) (B6: n=6, Prf^KO^: n=5, MHC-II^KO^: n=4, 3 independent experiments). *** P<0.001 (Mann-Whitney unpaired t-test). (**d**) Gate for CD45.1^+^TCRβ^+^CD8^+^ cells in infected spleen from BM chimeras. (**e**) Virus-specific T cells in mixed BM chimeras. H-2D^b^/GP33 vs CD44 profile of B6-origin CD8 T cells (CD45.1^+^TCRβ^+^) in the spleens from infected (day 8) or uninfected mixed BM chimeras. Numbers in profiles indicate frequency of cells in each box (day 8, n=3-4/group, 2 independent experiments). (**f**) CD44 vs IFN-γ profile of B6-origin CD8 T cells (CD45.1^+^TCRβ^+^) in the spleens from infected (day 8) or uninfected mixed BM chimeras. Splenocytes were stimulated with or without GP33 peptide. Numbers in profiles indicate frequency of cells in each box (n=3-4/group, 2 independent experiments).

Finally, to assess the in vivo functionality of CD4/MHC-II cytotoxic T cells, we examined responses of FlipFlop mice to acute infection with lymphocytic choriomeningitis virus Armstrong strain (LCMV-Arm)^45^. B6 mice successfully cleared the virus within 8 days, but FlipFlop mice failed to clear the virus, as revealed by the persistence of viral proteins in the serum and in the spleen (Fig. 6a,b). Nevertheless, both FlipFlop and B6 mice generated virus-specific cytotoxic T cells during virus infection (Fig. 6c). However, virus-specific T cells in FlipFlop mice were CD4 cytotoxic T cells that bound MHC-II tetramers composed of I-A^b^ + gp66 virus peptide, whereas virus-specific T cells in B6 mice were CD8 cytotoxic T cells that bound MHC-I tetramers composed of H-2D^b^ + gp33 virus peptide. FlipFlop virus-specific CD4 T cells expressed KLRG1 as did B6 CD8 T cells, confirming they were terminally differentiated cytotoxic T cells^46^ despite failing to provide protection against virus infection (Fig. 6c and Extended Data Fig. 7a).

Because virus-specific cytotoxic T cells generated in FlipFlop mice were CD4/MHC-II-specific, we wondered whether they might eliminate MHC-II^+^ virus-presenting cells and actively interfere with propagation of antiviral immune responses. To assess this possibility, we constructed mixed BM chimeras with FlipFlop and B6 BM cells which would contain B6-origin cytotoxic T cells that are capable of clearing infectious virus (Extended Data Fig. 7b). Remarkably, FlipFlop-origin cells did in fact prevent viral clearance in infected chimeras (Fig. 6d compare groups 1 and 2). Moreover, interference with viral clearance was specifically mediated by FlipFlop-origin cytotoxic T cells because the interference did not occur (that is, the virus was cleared) when FlipFlop-origin T cells were derived from FlipFlop.Prf^KO^ BM cells lacking the cytolytic protein Perforin (Prf)^30,47^ (Fig. 6d compare groups 2 and 3).

Viral clearance in B6 mice is mediated by CD8 cytotoxic T cells, as shown by the fact that B6 and MHC-II^KO^ mice clear virus whereas Prf^KO^ mice fail to clear virus (Extended Data Fig. 7c)^45,47^. Consequently, to determine whether FlipFlop CD4 cytotoxic T cells impaired the expansion of B6 virus-specific CD8 T cells in chimeras, we quantified the number of B6-origin virus-specific CD8 T cells (CD45.1) in their spleens (Fig. 6e). We found in chimeras with FlipFlop-origin cells that the number of B6-origin virus-specific CD8 T cells was markedly reduced, but their number was significantly higher (*P* < 0.05) when the FlipFlop-origin cells lacked Prf (Fig. 6e compare groups 1 and 2, and groups 2 and 3, and Extended Data Fig. 7d,e). These results reveal that the reduction in B6-origin virus-specific CD8 T cells was caused by FlipFlop-origin cytotoxic T cells (Fig. 6e compare groups 2 and 3, and Extended Data Fig. 7d,e). We confirmed these findings by measuring IFNγ production as the response of B6-origin (CD45.1) CD8 T cells to in vitro stimulation with MHC-I-specific viral peptide (Fig. 6f and Extended Data Fig. 7f). We found that IFNγ responses were significantly reduced when B6-origin virus-specific CD8 T cells were from chimeras containing FlipFlop-origin cells, and the responses were fully restored when the FlipFlop-origin cells lacked Prf (Fig. 6f and Extended Data Fig. 7f). Thus, CD4/MHC-II cytotoxic T cells which can target MHC-II^+^ virus-presenting cells actively inhibit in vivo expansion and propagation of protective virus-specific CD8 cytotoxic T cells.

Finally, if CD4/MHC-II cytotoxic T cells targeted MHC-II^+^ virus-presenting cells in vivo, they might also interfere with virus-specific B cell responses. In fact, while the total number of B6-origin B cells in chimeras was not affected by FlipFlop cells, we found that FlipFlop cells reduced the number of B6-origin GC B cells generated in virus-infected chimeras, consistent with the loss of MHC-II^+^ virus-presenting cells (Fig. 6g). Thus FlipFlop-origin CD4/MHC-II cytotoxic T cells do function in vivo but they do not protect against virus infection, partly because they cannot eliminate MHC-II^–^ parenchymal cells that are infected with virus and partly because they actively inhibit propagation of protective antiviral immune responses that are dependent on MHC-II^+^ virus-presenting cells.

## Discussion

The present study documents that T cell lineage fate is determined by *Cd4* and *Cd8* coreceptor gene loci that regulate the kinetics and duration of TCR signaling during positive selection, invalidating coreceptor signal-strength as the basis of T lineage determination. Switching coreceptor proteins encoded in *Cd4* and *Cd8* gene loci reverses the T cell immune system to contain CD4/MHC-II cytotoxic T cells and CD8/MHC-I helper T cells. Thus, both CD4 and CD8 coreceptor proteins promote helper T cell generation when encoded in *Cd4* but promote cytotoxic T cell generation when encoded in *Cd8*—explaining why helper-lineage T cells invariably express *Cd4*-encoded coreceptor proteins and cytotoxic-lineage T cells invariably express *Cd8*-encoded coreceptor proteins. We also documented that *Cd4* and *Cd8* gene loci dictate the duration of coreceptor-dependent TCR signaling during positive selection, with *Cd4*-encoded coreceptors promoting long-duration TCR signaling to induce helper-lineage fate and *Cd8*-encoded coreceptors promoting short-duration TCR signaling and inducing cytotoxic-lineage fate. Finally, reversed-function T cells fail to promote protective immunity, which explains why evolution fixed the particular coreceptor proteins that *Cd4* and *Cd8* gene loci encode in all surviving species.

The molecular basis for lineage determination during positive selection has remained an issue of contention, with lineage choice attributed to either TCR and coreceptor signaling strength or signaling duration^13,21,23^. FlipFlop mice with switched coreceptor proteins clearly distinguished TCR signal strength from TCR signal duration because weakly signaling CD8 coreceptors were controlled by *Cd4* gene loci, which promote long-duration TCR signaling and strongly signaling CD4 coreceptors were controlled by *Cd8* gene loci, which promote short-duration TCR signaling. Thus, CD8/MHC-I signaling was weak but of long duration, and CD4/MHC-II signaling was strong but of short duration. That CD8/MHC-I signaling generated CD8 helper T cells and CD4/MHC-II signaling generated CD4 cytotoxic T cells reveals that T cell lineage fate is not determined by TCR signaling strength but is determined by TCR signaling duration during positive selection.

It has been a classical paradigm of T cell immunology that TCR specificity dictates T cell function in the thymus, so that MHC-II-specific TCRs select helper T cells and MHC-I-specific TCRs select cytotoxic T cells^12^. The present study overturns this classical paradigm and significantly alters the understanding of why T cell specificity and function are linked during positive selection in the thymus. As shown in this study, MHC-II-specific TCR signaling generates helper T cells only when MHC-II-specific CD4 coreceptor proteins are encoded in *Cd4* gene loci; MHC-I-specific TCR signaling generates cytotoxic T cells only when MHC-I-specific CD8 coreceptors are encoded in *Cd8* gene loci. Thus, *Cd4* and *Cd8* gene loci determine helper and cytotoxic T lineage fates, respectively, and determine which coreceptor protein each T cell type expresses. Because functional T cells must express TCR and coreceptors with matching MHC specificities to transduce intracellular signals^33,48,49^, the MHC specificity of helper and cytotoxic T cells depends on which coreceptor protein *Cd4* and *Cd8* gene loci respectively encode.

Because FlipFlop mice contain a reversed T cell immune system, they revealed unique T cell subsets that have not previously been known. For example, FlipFlop mice generated CD8 T_reg_ and CD8 T_FH_ cells. T_reg_ cells in WT mice are almost exclusively CD4/MHC-II T cells, which has been interpreted as indicating a unique role in thymic T_reg_ generation for strongly signaling CD4 coreceptors and MHC-II-dependent ligands^37^. However, FlipFlop mice contained T_reg_ cells that were CD8/MHC-I helper-lineage T cells and which were as effective as WT T_reg_ cells in mediating self-tolerance and suppressing autoimmune responses. Thus, neither the generation nor function of T_reg_ cells uniquely depends on either CD4 coreceptor proteins or MHC-II-dependent self-ligands. Similarly, T_FH_ cells in WT mice are almost exclusively CD4/MHC-II T cells^41^. However, in vivo immunization of FlipFlop mice with soluble antigen revealed that CD8/MHC-I helper T cells become T_FH_ cells by interacting with DCs capable of cross-presenting peptides of soluble antigens onto MHC-I complexes^43,44^. Interestingly, CD8/MHC-I TFH cells are incapable of mediating cognate interactions with antigen-specific B cells, likely because B cells cannot cross-present peptides of soluble antigens onto MHC-I complexes. As a result, CD8/MHC-I T_FH_ cells fail to promote Ig class switching or the production of protective IgG antibodies. To further assess immune protection against viral pathogens, we examined the ability of FlipFlop mice to clear viral infection. Somewhat surprisingly, FlipFlop mice were unable to clear virus despite generating virus-specific cytotoxic T cells. Failure to clear virus was shown to be partly due to the fact that their virus-specific cytotoxic T cells were CD4/MHC-II-specific and actively interfered with antiviral immune responses by their elimination of MHC-II^+^ virus-presenting cells. Thus, reversed function CD8/MHC-I helper and CD4/MHC-II cytotoxic T cells fail to provide protective immunity against viral infections. Because protective immunity is necessary for species survival, our findings provide an evolutionary explanation for why *Cd4* gene loci encode MHC-II-specific CD4 coreceptors and *Cd8* gene loci encode MHC-I-specific CD8 coreceptors in all surviving species.

In conclusion, the present study has established that the functional polarity of the T cell immune system is determined by the MHC specificity of coreceptor proteins that *Cd4* and *Cd8* gene loci encode, and that in vivo protective immunity strictly requires the *Cd4* gene to encode an MHC-II-specific coreceptor protein and the *Cd8* gene to encode an MHC-I-specific coreceptor protein. In addition, this study has established that thymocyte lineage fate is determined by *cis*-regulatory elements in *Cd4* and *Cd8* gene loci that regulate the kinetics of coreceptor protein expression and the duration of TCR signaling during positive selection so that *Cd4*-encoded coreceptor proteins promote helper-lineage fate and *Cd8*-encoded coreceptor proteins promote the cytotoxic-lineage fate—regardless of the coreceptor protein each gene locus encodes.

## Methods

### Mice

C57BL/6 (CD45.1 and CD45.2) (B6) mice were obtained from Charles River Laboratory. BALB/c, CB6, β2m^KO^, Scurfy and Perforin^KO^ mice were purchased from The Jackson Laboratory and maintained in our own animal colony. MHC-II^KO^, Runx3d-YFP^50^, ThPOK-GFP^51^, and Foxp3-GFP knock-in (KI)^52^ mice were maintained in our own animal colony, as were OT-I.Rag2^KO^ and OT-II.Rag2^KO^ mice^53,54^. The mice were housed on a 12-h light–dark cycle at 20–26 °C with 30–70% humidity. All mice were analyzed without randomization or blinding. All mice analyzed were 6–12 weeks of age and both sexes were used unless mentioned otherwise in the manuscript. All animal experiments were approved by the National Cancer Institute Animal Care and Use Committee and were maintained in accordance with US National Institutes of Health guidelines.

### FlipFlop mice

FlipFlop mice with *Cd4* and *Cd8α* gene loci encoding the opposite coreceptor proteins were generated by mating mice with altered *Cd8* gene loci together with mice with altered *Cd4* gene loci. Mice whose *Cd8a* gene loci had been altered to encode CD4 coreceptor proteins (*Cd8*^CD4^) were previously reported and named 4in8 (ref. ^25^), whereas mice whose *Cd4* gene loci were altered to encode CD8 coreceptor proteins (*Cd4*^CD8^) needed to be constructed and were named 8in4 to indicate ‘CD8 encoded in *Cd4* gene loci’. For construction of 8in4 mice, we designed a gene-targeting vector and inserted it into the *Cd4* gene of B6 embryonic stem cells by homologous recombination (Extended Data Fig. 1a). The altered *Cd4*^CD8^ gene locus produces CD8.1αβ complexes that could be distinguished from B6-origin CD8.2αβ complexes. Because *Cd4* and *Cd8* gene loci are closely linked on chromosome 6, a genetic crossover event was necessary to generate a chromosome 6 allele containing both *Cd4*^CD8^*Cd8*^CD4^ altered gene loci, and we intercrossed mice with a crossover allele on one copy of chromosome 6 to generate FlipFlop mice with *Cd4*^CD8^*Cd8*^CD4^ crossover alleles on both copies of chromosome 6 (Fig. 1a right).

### Mixed bone marrow chimeras

T cell-depleted bone marrow cells from both CD45.1 and CD45.2 donor mice were mixed at a 1:1 ratio and 8 × 10^6^–10 × 10^6^ cells were injected intravenously into irradiated (950 rad) host B6 (CD45.1) mice. Chimeras were analyzed at 8–10 weeks after reconstitution.

### Cell preparation and flow cytometry

Cells were first incubated with anti-FcR (2.4G2, dilution 1:250) and stained with fluorescence-conjugated antibodies at 4 °C in HBSS supplemented with 0.5% BSA and 0.5% NaN_3_. Staining of CCR7 (dilution 1:50), CXCR5 (dilution 1:100) and MHC/viral peptide tetramers (H-2D^b^/GP33; dilution 1:500, I-A^b^/GP66; dilution 1:200) was performed at 37 °C for 30 minutes prior to staining with other antibodies. Staining of TCR-Vβs was performed using an anti-mouse TCR-Vβ screening panel (BD Biosciences). NP-binding GC B cells were stained using NP-PE (Biosearch, dilution 1:100). For intracellular staining of transcription factors and cytokines, cells were fixed and permeabilized with the Foxp3 Staining Buffer Set (Thermo Fisher Scientific) or BD Cytofix/Cytoperm Fixation/Permeabilization Kit (BD Biosciences), and then were stained with fluorescence-conjugated antibodies at 4 °C. ThPOK (2.5 μl) and Runx3 (5 μl) were stained for 1 hour at 4 °C. For cytokine staining, cells were stimulated at 37 °C with GP33 peptide (2 μg/ml, Anaspec) for 2 hours and added Golgi Stop (BD Biosciences) for 3 hours prior to staining. Stained cells were analyzed on a FACS LSRII or FACS Fortessa flow cytometer (Becton Dickinson). Electronic cell sorting was performed on a FACSAria II. Dead cells were excluded by forward light-scatter gating and staining with propidium iodide or LIVE/DEAD Fixable Aqua Dead Cell Stain Kit (Thermo Fisher Scientific) for fresh and fixed staining, respectively. Data were analyzed with EIB-Flow Control software developed at the US National Institutes of Health or FlowJo software (TreeStar). Antibodies in the following dilution were used: FAS (dilution 1:200), TCRβ (dilution 1:200), CD25 (dilution 1:100), GL3 (dilution 1:100), CD69 (dilution 1:200), CD5 (dilution 1:200), CTLA-4 (dilution 1:50), GL7 (dilution 1:800), Bcl6 (5 μg), CD8α (dilution 1:200), CD8β.2 (dilution 1:200), CD24 (dilution 1:200), CD44 (dilution 1:200), CD45.1 (dilution 1:200), CD45.2 (dilution 1:200), IFNγ (dilution 1:100), Nrp-1 (dilution 1:100), CD4 (dilution 1:200), Vα2 (dilution 1:100), Foxp3 (dilution 1:50), Helios (5 μl), B220 (dilution 1:200), ICOS (dilution 1:100), CD40L (dilution 1:50), and KLRG1 (dilution 1:100).

### Quantitative analysis of CD4 and CD8 surface expression

Thymocytes (1 × 10^6^) were incubated with 1 μg of non-conjugated anti-CD4 (GK1.5 rat IgG2b) or anti-CD8α (53-6.7 rat IgG2b) antibodies for 6 hours at 4 ^o^C. After being washed twice, they were incubated with 0.5 μg of FITC-conjugated anti-rat IgG antibody for 40 minutes at 4 ^o^C. After being washed twice, expression of FITC was analyzed by flow cytometry.

### Intracellular calcium mobilization

Thymocytes (2 × 10^6^) were loaded with the calcium-sensitive dye Indo-1 (1.8 mM, Thermo Fisher Scientific) at 31 °C for 30 minutes and then coated with anti-TCRβ (H57, 2 μg), CD4 (GK1.5, 0.5 μg), and CD8α (53-6.7, 0.5 μg) biotin-conjugated antibodies together with anti-CD4 FITC (RM4-4), anti-CD8β PE, and anti-CD69 APC-conjugated antibodies (dilution 1:200) at 4 °C for 40 min. Coated cells were kept at 4 °C until 2 min prior to stimulation, when cells were warmed and applied to the flow cytometer. Antibody crosslinking was induced with avidin (4 μg/ml), and data acquisition was recorded for 4 minues. Intracellular calcium concentrations were determined by the ratio of Indo-1 fluorescence at 405 versus 510 nm on CD69^–^CD4^+^CD8β^+^ thymocytes.

### In vitro stimulation of LN T cells

T cells were purified from LN with Pan T cells Isolation Kit (Miltenyi Biotec). LN T cells were stimulated with or without plate-bound anti-TCRβ (1 μg/ml) and anti-CD28 (1 μg/ml) antibodies for 24 hours at 37 °C. Cultured cells were stained and analyzed by flow cytometry for CD40L and CD69 expression.

### H&E stain and immunofluorescence staining

Indicated tissues were fixed with 10% PFA for paraffin sections or frozen with OCT compound (Sakura Finetek) and sectioned into 6-μm slices. Paraffin or frozen sections were used for H&E stain (Histoserv). For immunofluorescence staining, frozen sections were stained with indicated antibodies in Ca/Mg-free HBSS supplemented with 1% BSA at 20–26 °C for 2 hours. After washing, the sections were stained with fluorescence-conjugated antibodies (dilution 1:200) at 20–26 °C for 40 minutes. After washing, stained sections were mounted with Prolong diamond Antifide mountant (Thermo Fisher Scientific) and sealed with cover glasses. Images were acquired with Nikon CSU-W1 Spinning Disk Confocal Microscope (Nikon) and analyzed with Image J (National Institutes of Health). Anti-LCMV nucleoprotein (NP) antibody (VL-4; Bio X Cell) was labeled with Alexa Fluor 647 using a labeling kit (Thermo Fisher Scientific) and used at dilution 1:500. The following antibodies were used: anti-GL7 Alexa Fluor 488 (dilution 1:50), CD3 biotin (dilution 1:50), IgD Pacific Blue (dilution 1:50), anti-rat cytokeratin 8 (dilution 1:200), anti-rabbit cytokeratin 14 (dilution 1:100), streptavidin Alexa Fluor 568, goat anti-rat Alexa Fluor 546, goat anti-rabbit Alexa Fluor 488.

### Immunoprecipitation and immunoblotting

Cell lysates were prepared in lysis buffer (10 mM Tris-HCl (pH 7.4), 140 mM NaCl, 2 mM EDTA, 0.5% NP-40, and 1× protease inhibitor cocktail (Sigma)). The lysates were immunoprecipitated with anti-CD4-biotin (GK1.5; BD Pharmingen 7.5 μg) or CD8α-biotin (53-6.7; BD Pharmingen, 7.5 μg) antibodies together with streptavidin beads (Thermo Fisher Scientific) for 2 hours at 4 °C. Immunoprecipitants and cell lysates were resolved by SDS–PAGE under non-reducing conditions. The proteins were transferred to nitrocellulose membranes (Millipore Sigma) and incubated with anti-Lck (3A5; Santa Cruz, dilution 1:1,000)), followed by incubation with horseradish peroxidase (HRP)-conjugated anti-mouse IgG antibody (Thermo Fisher Scientific, dilution 1:2,500). Reactivity was revealed by enhanced chemiluminescence (Western Lightning ECL, Perkin Elmer). The intensity of the bands was quantified using Image Studio (LI-COR Biosciences).

### RT–qPCR analysis

Total RNA was isolated with RNeasy Plus Mini Kit (Qiagen), and cDNA was prepared with superscript III First-Strand Synthesis System for RT–PCR kit (Invitrogen). RT–qPCR was done with TaqMan PCR system (Thermo Fisher Scientific) or QuantiTect SYBR Green PCR system (Qiagen). TaqMan probes for Zbtb7b (ThPOK; Mm00784709_s1), Cd40lg (CD40L; Mm00441911_m1), Gata3 (Mm00484683_m1), Eomes (Mm01351985_m1), Prf1 (Perforin; Mm00812512_m1), and Rpl13a (Mm01612986_gH) were from Thermo Fisher Scientific. The primer sequences for SYBR green PCR system were as follows: Runx3d forward (5′- GCGACATGGCTTCCAACAGC-3′) and reverse (5′-CTTAGCGCGCTGTTCTCGC-3′); Rpl13a forward (5′-CGAGGCATGCTGCCCCACAA-3′) and reverse (5′-AGCAGGGACCACCATCCGCT-3′). Samples were analyzed on QuantStudio 6 Flex Real-time PCR System (Applied Biosystems). Gene expression values were normalized to those of Rpl13a expression in the same sample.

### RNA-sequencing analysis

CD4 and CD8 T cells were electronically sorted from LN of B6 and FlipFlop mice. Total RNA was prepared from sorted cells with the RNeasy Plus Mini Kit (Qiagen). The quality of RNA was assessed by Bioanalyzer (Agilent), and RNA samples with an RNA integrity number >9 were used. The library was made by using the SMARTer Universal Low Input RNA Kit (Clontech) for sequencing. The sequencing was performed as paired-end 125 bp by using HiSeq2500 equipment (Illumina). Reads were aligned to the mouse genome (mm10) with STAR aligner. Differentially expressed genes (DEGs) were genes whose fold change was more than fivefold and *P* value was less than 0.05. Visualization of DEGs was shown in a heat map generated with Partek (Partek).

### In vitro T_reg_ suppression assay

CD25^+^Foxp3^+^ (GFP^+^) T cells were electronically sorted from LNs of B6 Foxp3KI (CD4 T) and FlipFlop Foxp3KI (CD8 T) mice by flow cytometry. Sorted cells were cultured with CD4^+^CD25^−^ cells and irradiated (2,000 rad) splenocytes from B6 Foxp3KI mice in the presence of anti-CD3 antibody (145-2C11; BD Pharmingen, 1 μg/ml) at 37 °C for 3 days. After the culture, [^3^H]thymidine (400 μCi/ml) was added and incubated for 6 hours at 37 °C. [^3^H]thymidine incorporation was measured with MicroBeta counter.

### Skin allograft rejection

Tail skins prepared from BALB/c mice were grafted onto the flanks of host mice. Bandages were removed at day 7. Grafts were inspected every 1–2 days and were considered to have been rejected when <20% of the graft remained^55^.

### Immunization and ELISA

Mice were immunized intraperitoneally (i.p.) with 100 μg NP-KLH (Biosearch Technologies) mixed with 50 % (vol/vol) imject Alum (Thermo Scientific). Serum and tissues were collected at the appropriate time after immunization. For analysis of NP-binding cells, cells were incubated with NP-PE (Biosearch Technology) and analyzed by flow cytometry. NP-specific antibodies were analyzed using ELISA. Forty-eight-well plates were coated with NP-BSA (Biosearch Technologies) at 4 °C overnight, followed by incubation with serially diluted serum at room temperature for 1 hour. After washing, HRP-conjugated goat anti-mouse IgM (dilution 1:1,000) or IgG1 antibodies (Southern Biotech, dilution 1:2,000) were added to plates and incubated at room temperature for 1 hour. The reaction was developed by incubation with ABTS Peroxidase Substrate (KPL) and was stopped by ABTS Peroxidase Stop Solution (KPL). Plates were analyzed at 405 nm with Fluostar Optima plate reader and software (BMG Labtech).

### LCMV infection and viral titer assay

Mice were infected intravenously (i.v.) with LCMV-Armstrong (2 × 10^6^ PFU/mouse), and their serum and tissues were collected at day 8 for analysis. Viral titer in the serum from infected mice was assessed using a modified focus-forming assay^56^. Diluted serum (1:100) was incubated with Vero cells (2.5 × 10^4^ cells/well) on a 24-well plate at 37 °C for 4 hours. Each well was subsequently overlaid with 0.5% methylcellulose and incubated at 37 °C for 48 hours. Cells were subsequently fixed with 2% formalin/formaldehyde for 30 minutes and then with 0.5% Triton-X for 20 minutes. Fixed cells were stained with anti-LCMV NP antibody (VL-4; Bio X Cell, 5 μg/ml) for 1 hour, followed by staining with anti-rat IgG HRP antibody (Jackson Immunoresearch, dilution 1:1,350) for 1 hour. LCMV foci were visualized using an ImmPACT DAB Peroxidase (HRP) Substrate kit (Vector Labs).

### Statistical analysis

Statistical analysis was performed with GraphPad Prism 8 software using the two-tailed unpaired *t*-test. For comparison of skin graft survival, a log-rank (Mantel–Cox) test was used. *P* values of <0.05 were considered significant. For comparison of viral titer, a Mann–Whitney unpaired *t*-test was used. No statistical methods were used to predetermine sample sizes but our sample sizes are similar to those reported in previous publications^11^.

### Reporting Summary

Further information on research design is available in the Nature Research Reporting Summary linked to this article.

## Online content

Any methods, additional references, Nature Research reporting summaries, source data, extended data, supplementary information, acknowledgements, peer review information; details of author contributions and competing interests; and statements of data and code availability are available at 10.1038/s41590-022-01187-1.

## Supplementary information

Reporting Summary

## Data Availability

RNA-sequencing data of CD4 and CD8 LN T cells from B6 and FlipFlop mice are deposited at GEO under accession no. GSE166296. Source data are provided with this paper.

## Source data

Source Data Fig. 3 Uncropped scans of western blots in Fig. 3d.
