# Supplementary material for: Reversal of the T cell immune system reveals the molecular basis for T cell lineage fate determination in the thymus
Source: Nat Immunol. 2022 Apr 29;23(5):731–42. doi: 10.1038/s41590-022-01187-1 (PMC9098387; doi:10.1038/s41590-022-01187-1)
Supplement: Supplementary file 1 — Reporting Summary [file 41590_2022_1187_MOESM1_ESM.pdf]

Corresponding author(s): Alfred Singer
Last updated by author(s): 3/1/2022

Reporting Summary

Nature Portfolio wishes to improve the reproducibility of the work that we publish. This form provides structure for consistency and transparency in reporting. For further information on Nature Portfolio policies, see our Editorial Policies and the Editorial Policy Checklist.

Statistics

For all statistical analyses, confirm that the following items are present in the figure legend, table legend, main text, or Methods section.

n/a	Confirmed	
☐	☒	The exact sample size (n) for each experimental group/condition, given as a discrete number and unit of measurement
☐	☒	A statement on whether measurements were taken from distinct samples or whether the same sample was measured repeatedly
☐	☒	The statistical test(s) used AND whether they are one- or two-sided<br>*Only common tests should be described solely by name; describe more complex techniques in the Methods section.*
☒	☐	A description of all covariates tested
☐	☒	A description of any assumptions or corrections, such as tests of normality and adjustment for multiple comparisons
☐	☒	A full description of the statistical parameters including central tendency (e.g. means) or other basic estimates (e.g. regression coefficient) AND variation (e.g. standard deviation) or associated estimates of uncertainty (e.g. confidence intervals)
☐	☒	For null hypothesis testing, the test statistic (e.g. F, t, r) with confidence intervals, effect sizes, degrees of freedom and P value noted<br>*Give P values as exact values whenever suitable.*
☒	☐	For Bayesian analysis, information on the choice of priors and Markov chain Monte Carlo settings
☒	☐	For hierarchical and complex designs, identification of the appropriate level for tests and full reporting of outcomes
☒	☐	Estimates of effect sizes (e.g. Cohen's d, Pearson's r), indicating how they were calculated

Our web collection on statistics for biologists contains articles on many of the points above.

Software and code

Policy information about availability of computer code

Data collection	LSRII, Fortessa, Aria (Becton Dickinson), Nikon CSU-W1 Spinning Disk Confocal Microscope (Nikon), QuantStudio 6 Flex Real-time PCR System (Applied Biosystems), HiSeq2500 equipment (Illumina), MicroBeta counter, Fluostar Optima plate reader (BMG Labtech).
Data analysis	EIB-Flow Control version 7.5.0.0 (NIH), FlowJo version 10 (TreeStar), Prism 8 (Graph pad software), STAR aligner version 2.5.0, Partek version 7 (Partek Inc), ImageJ version 1.53o (NIH), Image Studio version 5.2 (LI-COR Biosciences).

For manuscripts utilizing custom algorithms or software that are central to the research but not yet described in published literature, software must be made available to editors and reviewers. We strongly encourage code deposition in a community repository (e.g. GitHub). See the Nature Portfolio guidelines for submitting code & software for further information.

Data

Policy information about availability of data

All manuscripts must include a data availability statement. This statement should provide the following information, where applicable:

- Accession codes, unique identifiers, or web links for publicly available datasets
- A description of any restrictions on data availability
- For clinical datasets or third party data, please ensure that the statement adheres to our policy

RNA-sequencing data of CD4 and CD8 LN T cells from B6 and FlipFlop mice (GEO: GSE166296)

Field-specific reporting

Please select the one below that is the best fit for your research. If you are not sure, read the appropriate sections before making your selection.

☒ Life sciences ☐ Behavioural & social sciences ☐ Ecological, evolutionary & environmental sciences

For a reference copy of the document with all sections, see nature.com/documents/nr-reporting-summary-flat.pdf

Life sciences study design

All studies must disclose on these points even when the disclosure is negative.

Sample size	The minimum sample size was chosen to reach statistical significance compared to control mice.
Data exclusions	No data were excluded.
Replication	For all experiment, at least three replicates were analyzed in at least two independent experiments. The experimental findings were reliably reproduced.
Randomization	Animals were allocated to groups based on their genotype.
Blinding	No blinding was used as no subjective scoring methods were used.

Reporting for specific materials, systems and methods

We require information from authors about some types of materials, experimental systems and methods used in many studies. Here, indicate whether each material, system or method listed is relevant to your study. If you are not sure if a list item applies to your research, read the appropriate section before selecting a response.

Materials & experimental systems

n/a	Involved in the study	
☐	☒	Antibodies
☒	☐	Eukaryotic cell lines
☒	☐	Palaeontology and archaeology
☐	☒	Animals and other organisms
☒	☐	Human research participants
☒	☐	Clinical data
☒	☐	Dual use research of concern

Methods

n/a	Involved in the study	
☒	☐	ChIP-seq
☐	☒	Flow cytometry
☒	☐	MRI-based neuroimaging

Antibodies

Antibodies used

Antibodies (clone, catalog number, RRID)
BD: FAS PE-Cy7 (Jo2, Cat# 557653; RRID: AB_396768), CXCR5 Biotin (2GB, Cat# 551960; RRID: AB_394301), TCRb (H57-597, Cat# 553166; RRID: AB_394679), TCRb FITC (H57-597, Cat# 553171; RRID: AB_394683), TCRb Biotin (H57-594, Cat# 553169; RRID: AB_394680) CD25 Biotin (Cat# 553070; RRID: AB_394602), CD25 PE (Cat# 553866; RRID: AB_395101), GL3 FITC (Cat# 553177; RRID: AB_394688), CD69 PE (H1.2F3, Cat# 553237; RRID: AB_394276), CD69 Biotin (H1.2F3, Cat# 553235; RRID: AB_394724), CD5 PE (53-7.3, Cat# 553023; RRID: AB_394561), ThPOK Alexa Fluor 647 (2POK, Cat# 565500; RRID: AB_2739268), Runx3 PE (R3-5G4, Cat# 564814; RRID: AB_2738969), CTLA-4 PE (UC10-4F10-11, Cat# 553720; RRID: AB_395005), GL7 FITC (GL7, Cat# 553666; RRID: AB_394981), Bcl6 PE (K112-91, Cat# 561522; RRID: AB_10717126), CD4 Biotin (GK1.5, Cat# 553728; RRID: AB_395012), CD8a Biotin (53-6.7, Cat# 553029; RRID: AB_394567), CD3e (145-2C11, Cat#: 553057; RRID: AB_394590), CD28 (37.51, Cat# 553294; RRID:AB_394763).
Bio X Cell: LCMV nucreoprotein (VL-4, Cat# BE0106; RRID: AB_10949017), TCR Vb screening panel (Cat# 557004; RRID: AB_647180).
Biolegend: CD8a Alexa Fluor 594 (53-6.7, Cat# 100758; RRID: AB_2563237), CD8b.2 Pacific blue (53-5.8, Cat# 140414; RRID: AB_10641278), CD24 Pacific blue (M1/M9, Cat# 101820; RRID: AB_572011), CD44 Pacific blue (IM7, Cat# 103020; RRID: AB_493683), GL7 Pacific blue (GL7, Cat# 144614; RRID: AB_2563292), PD-1 PE-Cy7 (29F.1A12, Cat# 135216; RRID: AB_10689635), CD45.1 PE (A20, Cat# 110708; RRID: AB_313497), CD45.1 FITC (A20, Cat# 110706; RRID: AB_313495), CD45.2 Alexa Fluor 594 (Ly-5.2, Cat# 109850; RRID: AB_2629589), CD45.2 FITC (Ly-5.2, Cat# 109806; RRID: AB_313443), IFNg PE (XMG1.2, Cat# 505808; RRID: AB_315402), Cytokeratin 14 (Poly19053, Cat# 905301; RRID: AB_2565048), GL7 Alexa Fluor 488 (GL7, Cat# 144612; RRID: AB_2563285), CD3 Biotin (17A, Cat# 100244; RRID: AB_2563947), IgD Pacific blue (11-26c.2a, Cat# 405712; RRID: AB_1937244), CD69 APC (Cat# 104514; RRID: AB_492843).
Bio search: NP PE (Cat# N-5070-1).
Harlan: CD16/32 (2.4G2, Cat# G208312).
Jackson Immunoresearch: Goat anti-rat IgG HRP (Cat# 112-035-143; RRID: AB_2338138).
Millipore Sigma: Cytokeratin 8 (TROMA-1, Cat# MABT329).
Promega: Anti-mouse IgG (H+L) HRP (Cat# W4021).

R&D system: Nrp1 Biotin (Cat# BAF566; RRID: AB_356581).
Santa Cruz: Anti-LCK (3A5, Cat# sc-433; RRID: AB_627880).
Southern Biotech: Goat anti-mouse IgM HRP (Cat# 1020-05; RRID: AB_2794201), Goat anti-mouse IgG1 HRP (Cat# 1070-05; RRID: AB_2650509).
Thermo Fisher Scientific: CD4 PE-Cy7 (RM4-5, Cat# 25-0042-82; RRID: AB_469576), CD4 FITC (RM4-4, Cat# 11-0043-82; RRID: AB_464900), CD8a APC (5H10, Cat# MCD0805; RRID: AB_10375296), CD8a APC eFluor 780 (53-6.7, Cat# 47-0081-82; RRID: AB_1272185), CD8b PE (eBioH35-17.2, Cat# 12-0083-83; RRID: AB_657767), CD24 APC eFluor 780 (M1/69, Cat# 47-0242-82; RRID: AB_10853172), Va2 FITC (B20.1, Cat# 11-5812-82; RRID: AB_465259), CCR7 Biotin (4B12, Cat# 13-1971-85; RRID: AB_466642), CCR7 PE (4B12, Cat# 12-1971-83, RRID: AB_465905), Foxp3 PE (FJK-16s, Cat# 12-5773-82; RRID: AB_465936), Foxp3 eFluor 660 (FJK-16s, Cat# 50-5773-82; RRID: AB_11218868), Helios PE (22F6, Cat# 12-9883-42; RRID: AB_2572758), B220 PE (RA3-6B2, Cat# 12-0452-83; RRID: AB_465671), B220 APC Fluor 780 (RA3-6B2, Cat# 47-0452-82; RRID: AB_1518810), ICOS PE (7E.17G9, Cat# 12-9942-82; RRID: AB_466274), CD40L PE (MR1, Cat# 12-1541-82; RRID: AB_465887), KLRG1 APC eFluor 780 (2F1, Cat# 47-5893-82; RRID: AB_2573988), KLRG1 PE (2F1, Cat# 12-5893-82, AB_10596642), Streptavidin Alexa 594 (Cat# S11227), Mouse hematopoietic lineage antibody cocktail FITC (Cat# 22-7770-72; RRID: AB_2644066), Streptavidin Alexa 568 (Cat# S11226), Goat anti-rat IgG Alexa 546 (Cat# A11081; RRID: AB_2534125), Goat anti-rabbit IgG Alexa 488 (Cat# A11008; RRID: AB_143165).
NIH Tetramer Core facility: H-2Db/GP33 APC, I-Ab/GP66 PE

Validation

All antibodies are commercially available and have been validated by the manufacturer or in previous reports.

Animals and other organisms

Policy information about studies involving animals; ARRIVE guidelines recommended for reporting animal research

Laboratory animals

Both male and female mice were used and analyzed at age 6-10 weeks old.
FlipFlop mice were generated by crossing 4in8 (Adoro et al., 2012) and 8in4 (this study) mice.
C57BL/6 (CD45.1 and CD45.2) (B6) mice were obtained from Charles River Laboratory (Wilmington, MA). BALB/c, CB6, B2mKO, Scurfy and PerforinKO mice were purchased from The Jackson Laboratory (Bar Harbor, ME) and maintained in our own animal colony. MHC-IIKO, Runx3d-YFP, ThPOK-GFP, and Foxp3-GFP knock-in (KI), OT-I.Rag2KO, and OT-II.Rag2KO mice were maintained in our own animal colony.

Wild animals

No wild animals were used in this study.

Field-collected samples

No field collected samples were used in this study.

Ethics oversight

All animal experiments were approved by the National Cancer Institute Animal Care and Use Committee and were maintained in accordance with US National Institutes of Health guidelines.

Note that full information on the approval of the study protocol must also be provided in the manuscript.

Flow Cytometry

Plots

Confirm that:

☐ The axis labels state the marker and fluorochrome used (e.g. CD4-FITC).

☒ The axis scales are clearly visible. Include numbers along axes only for bottom left plot of group (a 'group' is an analysis of identical markers).

☒ All plots are contour plots with outliers or pseudocolor plots.

☒ A numerical value for number of cells or percentage (with statistics) is provided.

Methodology

Sample preparation

Single cell suspensions were prepared by gently tweezing the organs with forceps in cold HBSS supplemented with 0.5% BSA and 0.5% NaN3.

Instrument

LSRII, Fortessa, Aria (Becton Dickinson)

Software

EIB-Flow Control (NIH), FlowJo version 10 (TreeStar)

Cell population abundance

>95% on sorted cells, which was determined by flow cytometry analysis on post sorted cells.

Gating strategy

Live cells were defined by FSC gating and staining with propidium iodide or LIVE/DEAD Fixable Aqua Dead Cell Stain Kit (Thermo Fisher Scientific) for fresh and fixed staining, respectively.
All gating strategies are stated in the manuscript.

☒ Tick this box to confirm that a figure exemplifying the gating strategy is provided in the Supplementary Information.
