# Supplementary figures and images for: Reversal of the T cell immune system reveals the molecular basis for T cell lineage fate determination in the thymus
Source: Nat Immunol. 2022 Apr 29;23(5):731–42. doi: 10.1038/s41590-022-01187-1 (PMC9098387; doi:10.1038/s41590-022-01187-1)

## Figure 3d

Top panel was used for "IP: CD4" and "Lysate".

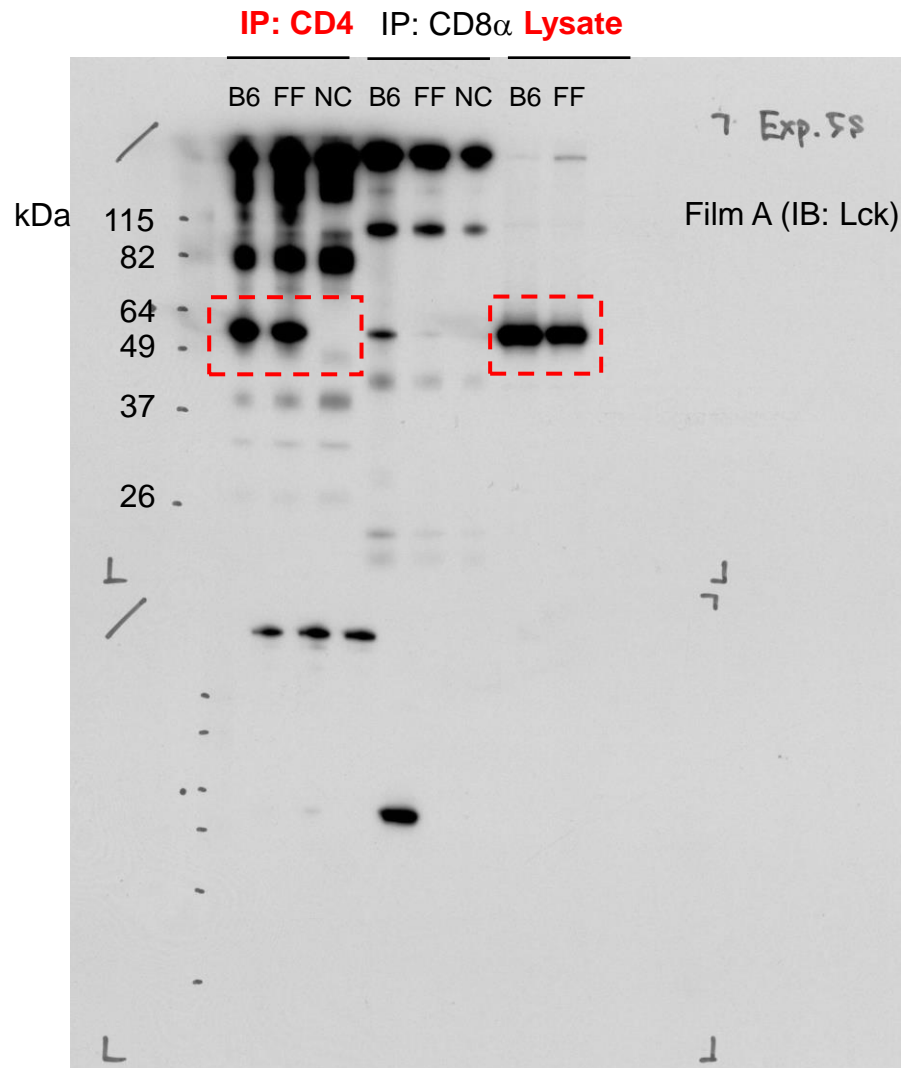

Top panel was used for "IP: CD8 $\alpha$ ".

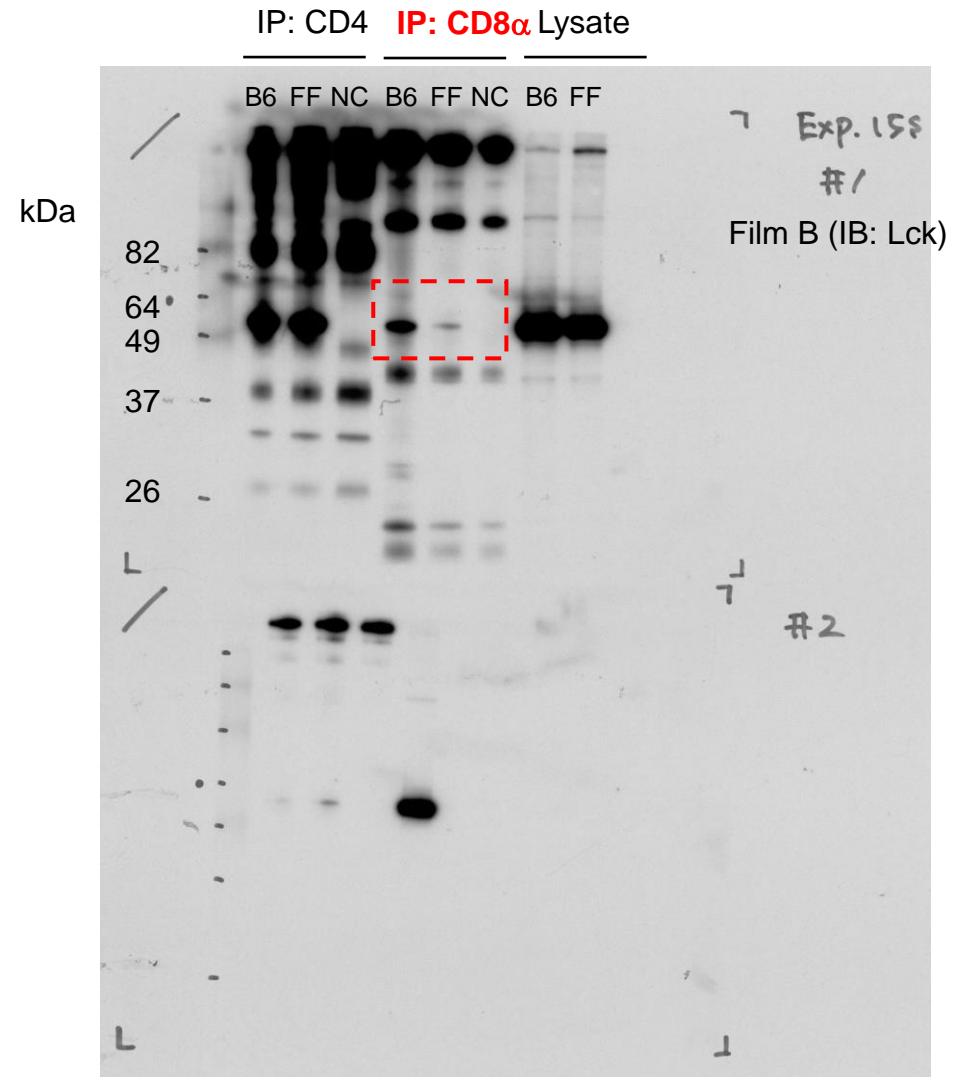

Supplement: Source Data Fig. 3 — Uncropped scans of western blots in Fig. 3d. [file 41590_2022_1187_MOESM2_ESM.pdf]
